# Molecular clock of HIV-1 envelope genes under early immune selection

**DOI:** 10.1186/s12977-016-0269-6

**Published:** 2016-06-01

**Authors:** Sung Yong Park, Tanzy M. T. Love, Alan S. Perelson, Wendy J. Mack, Ha Youn Lee

**Affiliations:** Department of Molecular Microbiology and Immunology, Keck School of Medicine, University of Southern California, 1450 Biggy Street, Los Angeles, CA 90089 USA; Department of Biostatistics and Computational Biology, University of Rochester, Rochester, NY 14642 USA; Theoretical Biology and Biophysics, Los Alamos National Laboratory, Los Alamos, NM 87545 USA; Department of Preventive Medicine, Keck School of Medicine, University of Southern California, Los Angeles, CA 90089 USA

**Keywords:** HIV-1, Envelope gene, Molecular clock, Mathematical model

## Abstract

**Background:**

The molecular clock hypothesis that genes or proteins evolve at a constant rate is a key tool to reveal phylogenetic relationships among species. Using the molecular clock, we can trace an infection back to transmission using HIV-1 sequences from a single time point. Whether or not a strict molecular clock applies to HIV-1’s early evolution in the presence of immune selection has not yet been fully examined.

**Results:**

We identified molecular clock signatures from 1587 previously published HIV-1 full envelope gene sequences obtained since acute infection in 15 subjects. Each subject’s sequence diversity linearly increased during the first 150 days post infection, with rates ranging from $1.54 \times 10^{ - 5}$ to $3.91 \times 10^{ - 5}$ with a mean of $2.69 \times 10^{ - 5}$ per base per day. The rate of diversification for 12 out of the 15 subjects was comparable to the neutral evolution rate. While temporal diversification was consistent with evolution patterns in the absence of selection, mutations from the founder virus were highly clustered on statistically identified selection sites, which diversified more than 65 times faster than non-selection sites. By mathematically quantifying deviations from the molecular clock under various selection scenarios, we demonstrate that the deviation from a constant clock becomes negligible as multiple escape lineages emerge. The most recent common ancestor of a virus pair from distinct escape lineages is most likely the transmitted founder virus, indicating that HIV-1 molecular dating is feasible even after the founder viruses are no longer detectable.

**Conclusions:**

The ability of HIV-1 to escape from immune surveillance in many different directions is the driving force of molecular clock persistence. This finding advances our understanding of the robustness of HIV-1’s molecular clock under immune selection, implying the potential for molecular dating.

**Electronic supplementary material:**

The online version of this article (doi:10.1186/s12977-016-0269-6) contains supplementary material, which is available to authorized users.

## Background

The molecular clock serves as a focal link between molecular evolution at a microscopic level and species evolution at a macroscopic level [1, 2]. The molecular clock hypothesis has been examined in a wide range of species both at the genomic and protein levels. Representative supporting data include (1) quantitative associations between amino acid sequence differences of homologous proteins and fossil-based divergence times of different organisms [3–5] and (2) linear relationships between the amount of nonsynonymous nucleotide substitutions and mammalian species divergence times [6].

Probing for an HIV-1 intrahost molecular clock is an important task because we can trace an infection back to transmission using sequences from a single time point if the molecular clock can be applied to an HIV-1 population within an infected individual. Accurately dating HIV-1 transmission is crucial for identifying risk behaviors that lead to transmission, monitoring prevention efforts, and informing when each immune response develops and matures. Estimates on the timing of infection can help us define immune correlates for protection using data from HIV-1 vaccine and prevention trials; for instance, knowledge of the time of HIV-1 acquisition will be important in determining the antibody titer threshold for protection in the Antibody Mediated Prevention (AMP) study [7]. Furthermore, the ability to molecularly date the HIV-1 gene pool expands the opportunity to determine HIV-1 incidence using recently developed genomic assays [8, 9].

The hypothesis that HIV-1 evolves in a clock-like manner has been tested; however, a consensus has not been reached. Rigorous statistical evaluations have been conducted on a diverse array of HIV-1 sequence data of different genomic regions, revealing both clock-like and non-clock-like behaviors [10–15]. The molecular clock hypothesis, while its existence itself is controversial, has been widely applied to estimate phylogenies and branching times of HIV-1 inter-host and intra-host populations: strict or relaxed molecular clocks were used to (1) date the ancestor of the main group of HIV-1 [16–18], (2) reconstruct the spread dynamics of HIV-1, estimating the location and timing of early transmission [19], and (3) quantify the intra-host HIV-1 envelope diversification rate in a range of $1.72 \times 10^{ - 5}$ per base per day to $4.32 \times 10^{ - 5}$ per base per day [20–23].

The HIV-1 gene population within an infected individual shows heavy selection signatures and fast-paced evolution due to a rapid turn-over rate and high mutation rate. Following transmission, an HIV-1 population evolves through the interplay of random mutations and immune selection in a complex setting of population growth and decline before reaching a stable virus load. This dynamic phase is a period of heightened immune selection pressure, which commences an evolutionary arms race between the virus and the immune system. Around 1 month post infection, the first CD8+ T cell responses targeting the founder viruses lead to rapid viral escapes with amino acid changes in CD8+ T cell epitope sequences at a rate as fast as 0.42 per day [24, 25]. This rate implies that a minor mutant present in 5 % of the total viral population could become the dominant lineage making up 95 % of the population in just 2 weeks. In the wake of the early CD8+ T cell responses, initial neutralizing antibody responses develop at around 3 months post infection, resulting in an ongoing pattern of viral escape and antibody evolution [26–28]. Understanding the effect of strong immune selection on HIV-1’s molecular clock is of interest as selection is often thought to be a rate-changing factor [2, 29], driving a genealogy to depart from that of random evolution by placing preference for particular lineages and perturbing the molecular clock.

In this study, we empirically and theoretically examine evidence of molecular clock conservation under selection by combining gene sequence data with mathematical models for HIV-1 evolution. We analyzed previously published HIV-1 envelope gene sequences collected from within 1 month of infection with sample intervals of days and weeks and traced HIV evolution at the onset of immune selection. Our primary goal is to examine whether a selection-induced heterogeneous phylogeny can conform to a strict molecular clock. By mathematically quantifying deviations from the molecular clock in an array of selection scenarios, we define conditions for the existence of a molecular clock.

## Results

**Fig. 1:**
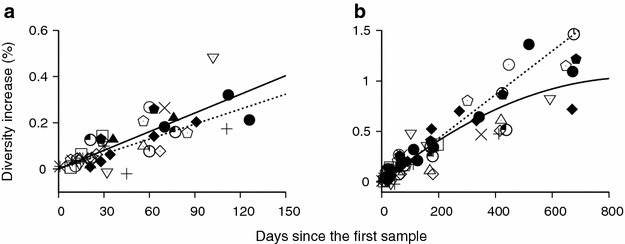
HIV-1 diversity dynamics of 15 subjects. **a** Diversity increase as a function of time for the first 150 days since the first sample for 15 HIV-1 infected individuals, CAP045, CH040, CH042, CH058, CH077, CH131, CH159, CH162, CH164, CH185, CH198, CH256, CH505, SUMA0874 and WEAU0578. A total of 1587 HIV-1 full envelope gene sequences were obtained from references [27, 30–32]. The population dynamics showed a statistically significant linear diversification rate of 2.69 (±0.29) × 10^−5^ per site per day (p < 0.0001). The *solid line* represents the fit of the mixed effects model and the *dotted line* represents the neutral diversification rate, $2.16 \times 10^{ - 5}$ per site per day [33–35]. **b** Diversity dynamics over 2 years since the first sample of the 15 subjects. The *solid curve* denotes the best-fit of the mixed effects model to these overall dynamics. Over the 2 year time frame, the envelope gene sequence population diversifies linearly at a rate of $2.30 \times 10^{ - 5}$ per site per day (p < 0.0001) and quadratically at a rate of $- 0.00122 \times 10^{ - 5}$ per site per day^2^ (p = 0.028). The *dotted line* again represents the neutral diversification rate, $2.16 \times 10^{ - 5}$ per site per day

We examined HIV-1 diversification patterns under immune selection from serial measures of HIV-1 envelope gene sequence diversity. We analyzed 1587 previously published HIV-1 whole envelope gene sequences obtained serially from 15 acutely infected individuals [27, 30–32]. Figure 1a plots HIV-1 envelope gene sequence diversity dynamics during the 150 days following the first sample. To avoid the uncertainty of when each subject’s first sample was taken, all subsequent data points are presented in terms of the increase in diversity and the time following the first sample.

**Fig. 2:**
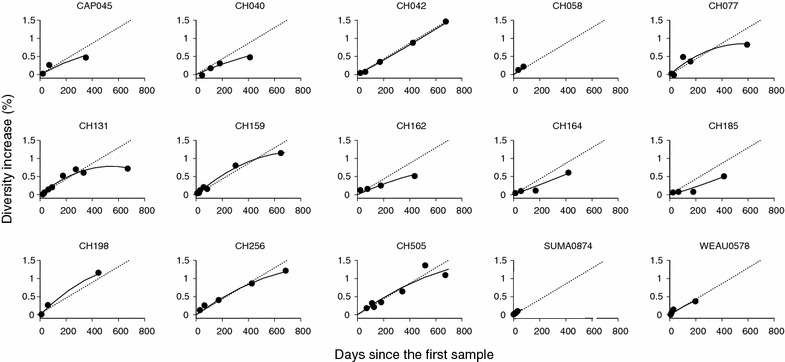
Individual HIV-1 diversity dynamics. Each of the 15 subjects’ diversity dynamics as a function of time over 2 years since the first sample. The *dotted line* of each panel indicates the neutral diversification rate, $2.16 \times 10^{ - 5}$ per site per day [33–35], and the *solid curve* denotes the best-fit of the mixed effects model. All 15 subjects show a statistically significant linear increase in diversity over time; subjects CH077, CH131, CH159 and CH505’s quadratic increases are statistically significant. The linear diversification rate for the first 150 days of each individual is listed in Table 1 and the linear and quadratic diversification rates over 2 years are listed in Additional file 1: Table S1

A mixed effects model was used to analyze the 15 subjects’ diversity dynamics over time (see “Methods”). At the population level, in the first 150 days following the first sample, the linear diversification of all 15 subjects’ HIV sequences was statistically significant (p < 0.0001), while quadratic attenuation was not (p = 0.76) (Fig. 1a). At the individual level, each subject’s sequence population showed statistically significant linear relationship (Table 1). This rate of linear diversification ranged from $1.54 \times 10^{ - 5}$ to $3.91 \times 10^{ - 5}$ per base per day with a population mean of 2.69 (±0.29) × 10^−5^ (Table 1), which is close to the HIV-1 diversification rate under the neutral evolution assumption (i.e. all HIV-1 infected cells produce the same number of secondary infected cells in a single replication cycle), $2.16 \times 10^{ - 5}$ per base per day [33–35]. This neutral evolution rate was approximated as $2\varepsilon /\tau$ with the viral generation time $\tau = 2$ days and HIV-1 single cycle base substitution rate $\varepsilon = 2.16 \times 10^{ - 5}$ per base per cycle [35]. We found that 12 out of the 15 subjects’ rates of diversification matched the neutral evolution rate (Table 1). We did not observe any differences in the linear diversification rate between males and females (p = 0.70, ANOVA), contradicting a recent study that reported a greater evolution rate in risk groups with a higher proportion of men [36]. We then traced HIV-1 diversity over a longer time frame of 2 years, as shown in Fig. 1b, a quadratic attenuation became significant at the population level (p = 0.028). Figure 2 plots each subject’s diversity dynamics over 2 years of infection with the best-fit of a mixed effect model (Additional file 1: Table S1). The quadratic leveling-off of diversity was significant over 2 years in 4 of the 15 subjects, CH077, CH131, CH159 and CH505 (see p values for each quadratic term in Additional file 1: Table S1). Our observation suggests that an intrahost HIV-1 population evolves in a clock-like manner close to the error rate of HIV-1 reverse transcriptase for the first 150 days following infection and starts to slowly level off afterwards.

**Table 1:** The rate of HIV-1 envelope gene sequence diversification with standard errors during the 150 days from the first sample in 15 subjects whose sequence data come from references [27, 30–32]

Subject	Rate of linear diversification with standard errors (× 10^−5^ per base per day)	p-value	p-value for departure from the linear neutral rate^a^	Rate of diversification in selection sites (× 10^−5^ per base per day)	Rate of diversification in non-selection sites (× 10^−5^ per base per day)
CAP045	3.24 ± 0.56	<0.0001	0.073	8.74 ± 57.8	3.04 ± 0.50
CH040	1.54 ± 0.39	0.0013	0.13	47.3 ± 35.9	1.16 ± 0.35
CH042	1.96 ± 0.61	0.0061	0.74	28.2 ± 65.8	1.48 ± 0.55
CH058	2.91 ± 0.51	<0.0001	0.16	633.9 ± 50.6	2.03 ± 0.46
CH077	3.91 ± 0.42	<0.0001	0.00097	91.2 ± 39.8	3.09 ± 0.38
CH131	2.21 ± 0.38	<0.0001	0.90	27.1 ± 35.6	1.80 ± 0.34
CH159	2.57 ± 0.42	<0.0001	0.34	22.2 ± 39.4	2.21 ± 0.37
CH162	2.46 ± 0.53	0.0004	0.57	78.0 ± 53.2	1.89 ± 0.47
CH164	2.26 ± 0.64	0.0033	0.88	43.4 ± 71.9	1.83 ± 0.58
CH185	1.86 ± 0.57	0.0053	0.61	47.2 ± 59.0	1.51 ± 0.51
CH198	3.57 ± 0.62	<0.0001	0.039	50.4 ± 68.4	3.09 ± 0.56
CH256	3.58 ± 0.58	<0.0001	0.028	57.3 ± 61.0	2.77 ± 0.52
CH505	2.30 ± 0.27	<0.0001	0.60	45.4 ± 23.7	1.57 ± 0.24
SUMA0874	2.85 ± 0.78	0.0027	0.39	706.8 ± 109.8	2.17 ± 0.70
WEAU0578	3.17 ± 0.79	0.0013	0.22	190.8 ± 113.8	2.39 ± 0.71
Population mean	2.69 ± 0.29	N/A	N/A	138.5 ± 61.7	2.13 ± 0.26

^a^Less than 0.05 implies a statistically significant deviation from the neutral evolution rate, 2.16 × 10^−5^ per site per day

**Fig. 3:**
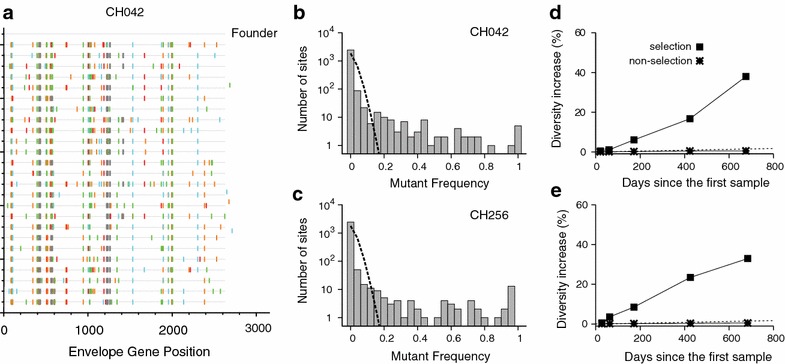
Diversity dynamics within selection sites and non-selection sites. **a** The highlighter plot (http://www.hiv.lanl.gov/content/sequence/HIGHLIGHT/highlighter_top.html) of subject CH042’s envelope gene sequences obtained 676 days after the first sample, along with the founder envelope sequence (*top*). Here the founder envelope sequence was inferred as the consensus sequence of subject CH042’s first sample at Fiebig stage I/II. The subsequent sequences show positions of mutations away from the founder sequence. **b** The mutant frequency distribution for subject CH042 in Ref. [32] (*grey bars*). Mutant frequency denotes the proportion of sequences from a particular time point that do not match the founder sequence nucleotide at a given site. The *dotted line* presents the best fit of the binomial distribution in Eq. (1) to subject CH042’s mutant frequency distribution. The threshold value of mutant frequency is determined when the tail of the fitted curve becomes less than 0.1; sites above this threshold of 0.2 were designated as putative selection sites. A total of 63 putative selection sites were identified. **c** The mutant frequency distribution of subject CH256 in Ref. [32] (*grey bars*) along with the best fit of the binomial distribution (*dotted line*). The threshold mutant frequency is determined as 0.2, where the tail of the binomial distribution becomes less than 0.1. The 53 sites with a mutant frequency above the threshold were regarded as putative selection sites. **d** The diversity dynamics of selection sites (*squares*) compared with those within non-selection sites (*asterisks*) for subject CH042. The slope of diversity at selection sites is around $28.2 \times 10^{ - 5}$ per base per day, which is approximately 19 times greater than that of non-selection sites for the first 150 days after the first sample. The *dotted line* presents the neutral rate of evolution, $2.16 \times 10^{ - 5}$ per site per day. **e** The diversity dynamics of selection sites (*squares*) compared with those of non-selection sites (*asterisks*) for subject CH256. The slope of diversity increase at selection sites was around 21 times greater than that at non-selection sites ($57.3 \times 10^{ - 5}$ vs. $2.77 \times 10^{ - 5}$ per base per day)

We next examined spatial patterns of mutations across the envelope gene. Figure 3a shows mutations away from the founder sequence along subject CH042’s envelope gene sequences; the locations of mutations are clustered on putative selection sites rather than randomly scattered. Previously, epitope mapping uncovered regions susceptible to immune selection, and thus viral escapes via mutation in these 15 subjects [27, 30–32]. Experimentally identifying all selection sites, however, was not feasible. Alternatively, a statistical approach can provide a comprehensive list of putative selection sites based on patterns in sequence samples. We defined putative selection sites as nucleotide positions showing more base substitutions from the founder nucleotide than would be expected to occur by chance in the absence of selection. To designate putative selection sites, we first measured the mutant frequency: the proportion of sequences at a given time that do not match the founder sequence at a particular nucleotide site. Figure 3b plots the mutant frequency distribution of all sites along 25 full envelope gene sequences obtained from subject CH042 at 676 days from the first sample date. In the absence of selection, the number of sequences, *k*, at a given time post infection, *t*, that do not match the founder sequence at a particular nucleotide site would follow a binominal distribution,

1 $$P(k,t) = \frac{{N_{S} !}}{{k!(N_{S} - k)!}}\left( {\frac{t}{\tau }\varepsilon } \right)^{k} \left( {1 - \frac{t}{\tau }\varepsilon } \right)^{{N_{S} - k}} ,$$

where $N_{S}$ is the number of sampled sequences, $\varepsilon$ is the HIV-1 single cycle base substitution rate and $\tau$ is the viral generation time [20–23]. The best fit of the binomial distribution to subject CH042’s mutant frequency distribution is presented by the dashed line in Fig. 3b. This fit defines a threshold mutant frequency such that sites exhibiting mutations from the founder sequence above the threshold mutant frequency are designated as putative selection sites.

Indeed, some statistically identified selection sites match experimentally confirmed peptides reactive to autologous CD8+ T cells, including VQKEYAFFYK (169–178) and QFRNKTIVF (gp160 352–361) [32]. Some of these selection sites we identified that are consistent with known CD8+ T cell epitopes are also restricted by the same HLA type for each individual. Additional file 1: Table S2 links, when applicable, each designated selection site to a known CD8+ T cell epitope in the Los Alamos National Laboratory HIV-1 Molecular Immunology Database (http://www.hiv.lanl.gov/content/immunology/maps/maps.html). On average, around 60 % of the statistically identified selection sites were located within known CD8 T cell epitope regions (Additional file 1: Table S1).

Clear immune selection signatures were visible when we compared diversity dynamics between selection and non-selection sites. Subject CH042’s sequence data showed that diversity increased more rapidly within designated selection sites than it did outside of them (Fig. 3d). The slope of the selection sites’ diversity increase was around 19 times greater than that of non-selection sites in subject CH042 over 150 days. We observed a similar pattern in subject CH256 (Fig. 3e) and all other subjects. Our mixed effects model estimated that, on average, selection sites diversify around 65 times more rapidly than non-selection sites during the first 150 days post infection (Table 1). Finding the majority of base substitutions in sites which cover between 0.12 and 3.14 % of the full envelope gene indicates that the capacity to select viral variants is highly concentrated within immune targeted sites.

While patterns of multiple mutant forms were prevalent in selection sites, some sites become homogeneous after the first 150 days since infection; for example, in 12 putative selection sites of subject CH131, the same single nucleotide replaced the founder nucleotide in all sequences at each respective site 670 days after the first sample date. In this subject, we observed a smaller increase in genetic diversity than predicted by the molecular clock (Fig. 2) due to these homogeneous selection sites. Some selection sites were homogeneous in four other subjects, CAP045, CH077, CH185, and CH256, showing deviations from the constant evolution rate caused by decreased diversity, as shown in Fig. 2. Nonetheless, hard sweep signatures are lacking within these five subjects because the frequency of heterogeneous selection sites remains substantial, ranging from 38.5 to 75.5 %; in the other 10 subjects, the homogeneous sites make up fewer than 20 % of all identified putative selection sites. While homogeneity creates notable deviations from clock-like evolution after the first 150 days post infection, it is most commonly not the primary determinant of HIV-1 selection patterns.

**Fig. 4:**
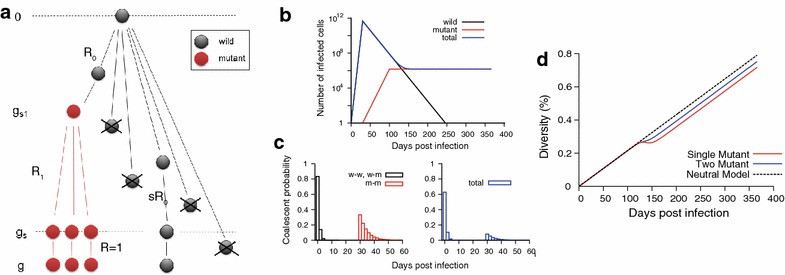
Modeling HIV-1 evolution under immune selection. **a** Schematic diagram of the HIV-1 selection model. The wild-type infected cell (*black*) produces $R_{0}$ offspring up to generation $g_{s1}$ and afterwards produces $sR_{0}$ offspring due to immune selection ($sR_{0} < 1$). One mutant-type infected cell (*red*) starts to produce $R_{1}$ secondary infected cells at generation $g_{s1}$. At the viral set point ($g \ge g_{s}$) the reproductive ratio of the mutant-type infected cells becomes equal to 1. **b** The total HIV-1 infected cell population level (*blue line*) as a function of time post infection. The infected cell population consists of the wild-type (*black*) and mutant-type infected cells (*red*). The model parameters are $R_{0} = 6$, $sR_{0} = 0.78$, $R_{1} = 1.5$, $g_{s1} = 15$ (30 days) and $g_{s} = 50$ (100 days). The HIV-1 single cycle mutation rate is $2.16 \times 10^{ - 5}$ per base per generation [33] and the generation time is 2 days [34]. The wild-type infected cell population grows exponentially and starts to decline at the emergence of selection pressure, approximately 1 month post infection [24, 28]. Subsequently, a mutant-type infected cell arises and replicates exponentially. **c** The coalescent time distribution of wild–wild virus pairs (*black bars*) and mutant–mutant pairs (*red bars*). The coalescent time distribution of wild-mutant pairs is the same as that of wild–wild pairs (*black bars*). The wild–wild and wild-mutant pairs most likely coalesce at the transmission point, while the most recent common ancestor of a mutant-type virus pair is the mutant-founder virus that appears at generation $g_{s1}$, i.e. 30 days post infection. When the wild-type virus population size is comparable with the mutant-type population size at 132 days post infection, the coalescent probability of any random viral pair including wild–wild, wild-mutant, and mutant–mutant pairs (*blue bars*) peaks at the transmission point (generation 0). **d** Diversity dynamics of the one-mutant model (*red line*), two-mutant model (*blue line*), and neutral model (*dotted black line*). The model parameters for the two-mutant model are *R*_0_ = 6, *sR*_0_ = 0.78, *R*_1_ = 1.5, *R*_2_ = 1.5, *g*_*s*1_ = 15 (30 days), *g*_*s*2_ = 16 (32 days), and *g*_*s*_ = 50 (100 days). The diversity dynamics of the two-mutant model more closely resemble the neutral model’s diversity dynamics than the one-mutant model’s

Next, we sought to understand the link between clock-like evolution and clustered mutation at immune selection sites, which were simultaneously observed in a considerable number of envelope gene sequences from the 15 individuals. We developed a model of HIV-1 gene evolution and within-host viral dynamics during the early phases of immune selection. As illustrated in Fig. 4a, in our model the founder lineage initially replicates in the absence of immune selection, producing *R*_0_ secondary infected cells from a single infected cell [30, 35]. Each replication cycle involves HIV-1 reverse transcriptase-mediated base substitution errors with rate *ε*. Departing from neutral evolution, at the onset of selection, at generation *g*_*s*1_, a single infected cell harboring an escape virus is assumed to arise and begin producing *R*_1_ daughter cells, while the replicative capacity of the wild-type infected cells is significantly compromised due to immune recognition, producing only *sR*_0_ daughters on average, with 0 < *sR*_0_ < 1. Thus, the selection coefficient of the wild-type virus relative to the escape mutant is *S* = 1 − (*sR*_0_/*R*_1_). During the viral decline phase, the wild-type population is rapidly cleared by immune selection while the proportion of the mutant-type population increases within the total population, leading to viral escape. After the viral set point is reached (generation *g*_*s*_ in Fig. 4a), all existing mutant-type infected cells are assumed to repopulate themselves without increasing the population size ($R_{1} = 1$). In this model, the total infected cell count and viral load, proportional to the former, mimic what is observed through the natural course of an HIV-1 infection—an exponential increase followed by a rapid decline, with a steady population level thereafter (Fig. 4b).

Despite modification of HIV-1 genealogy by immune selection, our model showed that any given wild-type virus pair most likely coalesces at the transmission point [Additional file 1: Eq. (S14)]. The genealogy of mutant-type pairs followed a neutral evolution scenario wherein all mutant descendants originate from a single mutant ancestor. As shown in Fig. 4c, the coalescence probability of the mutant-type pairs peaked when the first mutant virus appears and exponentially decreases afterwards [Additional file 1: Eq. (S17)]. Our calculation also showed that a wild-mutant virus pair most likely coalesces at the transmission point with the same trend as does a wild–wild pair [Additional file 1: Eq. (S20)]. The most recent common ancestor (MRCA) for both wild–wild and wild-mutant pairs was most likely the founder virus, whereas for mutant–mutant pairs it was most likely the first mutant virus. Therefore, the total population coalescence distribution depended on the ratio between the wild-type population and the mutant one. When the mutant population level was comparable to that of the wild-type, the coalescent profile peaked at the origin of an infection, as shown in Fig. 4c.

The coalescent profile of all virus pairs permits us to evaluate diversity dynamics, from which we can assess deviations from the molecular clock. The deviation from a constant molecular clock can be quantified as the difference between the sequence diversity and the reference neutral clock diversity. As detailed in Additional file 1, when the mutant population was prevalent at the viral set point, the clock deviation, $\Delta_{1}$, was approximated as

2 $$\Delta _{1} \simeq 2\varepsilon \left\{ {g_{s1} + \frac{1}{{R_{1} - 1}} - \frac{1}{{R_{0} - 1}}} \right\}$$

Here Eq. (2) indicates that the clock deviation is mainly affected by the time that the escape lineage arises (*g*_*s*1_); the later it appears, the greater the deviation. The replicative capacity of the mutant-type virus (*R*_1_) and wild-type virus (*R*_0_) also contribute to the clock deviation. On the other hand, the deviation does not depend on the selective disadvantage of the wild-type population (*s*). Likewise, the clock deviation is approximately constant regardless of when viruses are sampled (*g*) after the mutant lineage arises.

Our findings demonstrated that a fraction of selection sites showed an extreme level of diversity (Fig. 3), revealing the presence of multiple escape lineages. Accordingly, we generalized the model to more rigorously address how the clock deviation changes as more mutant lineages accrue. The generalized *N*-mutant model showed that all virus pairs except intra-mutant pairs (those within a single mutant lineage), including wild–wild, wild-mutant, and inter-mutant pairs, most likely coalesce at the founder virus [see Additional file 1: Eqs. (S66), (S69), and (S72)]. The deviation from the molecular clock decreases as more distinct mutant lineages appear, approximated as the single mutant model deviation divided by the number of mutant lineages, *N*,

3 $$\Delta _{N} \simeq \frac{{\Delta _{1} }}{N}.$$

This calculation indicates complete molecular clock conservation in the large *N* limit. For instance, Fig. 4d shows that when two mutant viruses appear, the clock deviation becomes half of that of the single mutant model. The conservation of the molecular clock in a selection-induced heterogeneous phylogeny can be understood from our demonstration that distinct mutant lineages most probably coalesce at the origin of an infection. As more distinct mutant lineages appear, the proportion of virus pairs coalescing to the initial transmission point increases within the total viral population, decreasing the deviation from the molecular clock. Ergo, even when the transmitted/founder lineage is entirely eliminated from the viral population, this clock property allows us to assess the time since infection based on sequences of escape mutant populations, permitting molecular dating of HIV-1 infections.

**Fig. 5:**
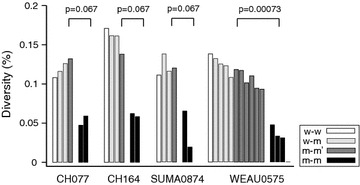
Virus pair diversity comparisons. Diversity of wild–wild virus pairs (*white bars*), wild-mutant pairs (*light grey bars*), virus pairs from different mutant lineages (*dark grey bars*), and virus pairs from a single mutant lineage (*black bars*). The sequence data from subject CH077, SUMA0874, and WEAU0575 were obtained from Ref. [30] and the sequence data from subject CH164 were obtained from Ref. [32]. The sample time point is 14, 56, 30, and 15 days after the first sample at Fiebig stage I/II for subject CH077, CH164, SUMA, and WEAU, respectively. Table 2 lists wild and mutant epitope sequences of each subject at a given time point. For example, subject CH077’s sample 14 days after the first sample had one wild type lineage with the epitope QFRNKTIVF and two mutant lineages with either the epitope QF**K**NKTIVF or QFRNK**A**IVF. Here, the wild-type lineage was designated in reference to the consensus sequence of the first time point sequences of each individual. The full envelope gene sequence diversity of mutant-type pairs from distinct lineages was comparable to that of the wild-type lineage. As predicted by our model, the diversity within each mutant lineage was considerably smaller than that of wild–wild pairs, wild-mutant pairs, and virus pairs from different mutant lineages (Wilcoxon–Mann–Whitney test, p = 0.067 for CH077, p = 0.067 for CH164, p = 0.067 for SUMA0874, and p = 0.00073 for WEAU0575)

In the presence of multiple escape lineages, our model predicted that the most probable coalescent point of any two distinct mutant lineages would be the same as that of wild-type virus pairs: the transmission point. We tested this prediction by comparing the diversity among mutant-type pairs from distinct lineages and the diversity among wild-type pairs. To clearly designate distinct mutant lineages, we selected four subjects whose envelope sequences at a chosen time showed the signature of escape within only one epitope. Table 2 lists the CD8+ T cell epitope sequences of both founder and mutant lineages for each of these four cases. Here, the wild-type lineage is designated in reference to the consensus sequence of the earliest time point sample of each individual. Each mutant lineage was grouped based on amino acid sequence variations within the CD8+ T cell epitopes. As in Table 2, more than one escape lineage derived from the same epitope existed in all four cases. As predicted by our model, the diversity among mutant-type pairs from distinct lineages and wild-type pairs are highly similar to one another (Fig. 5). Contrarily, the diversity within each mutant lineage is considerably smaller than that of the founder lineage (Fig. 5), which is in good agreement with the prediction that intra-mutant lineage pairs coalesce at a later generation than do wild-type lineage pairs.

**Table 2:** Founder and escape lineages of a given epitope from each subject’s single time point sequence data

Patient	Number of envelope sequences	Escape details
CH077		gp160 (352–361)
Founder-lineage	9	QFRNKTIVF
Escape-mutant 1	38	QF**K**NKTIVF
Escape-mutant 2	4	QFRNK**A**IVF
CH164		gp160 (2–10)
Founder-lineage	5	RVMETRRSW
Escape-mutant 1	10	RVM**K**TRRSW
Escape-mutant 2	8	RVM**G**TRRSW
SUMA0874		gp160 (744–753)
Founder-lineage	18	RSSRLVDGFL
Escape-mutant 1	6	RS**G**RLVDGFL
Escape-mutant 2	4	RSS**C**LVDGFL
WEAU0575		gp160 (31–39)
Founder-lineage	24	AENLWVTVY
Escape-mutant 1	18	AE**K**LWVTVY
Escape-mutant 2	7	**T**ENLWVTVY
Escape-mutant 3	5	**V**ENLWVTVY
Escape-mutant 4	4	A**K**NLWVTVY

The sequence data from subject CH077, SUMA0874, and WEAU0575 are obtained from Ref. [30] and the sequence data from subject CH164 are obtained from Ref. [32]. The sample time point is 14, 56, 30, and 15 days after the first sample at Fiebig stage I/II for subject CH077, CH164, SUMA, and WEAU, respectively

## Discussion

The present study examined the rate of HIV-1 envelope gene diversification within 15 individuals who were serially surveyed from the acute stage of infection. HIV-1 sequence diversity increased linearly for the first 150 days of infection with a population mean of $2.69 \times 10^{ - 5}$ per base per day. This clock-like evolutionary pattern showed variations in speed across the subjects, ranging from $1.54 \times 10^{ - 5}$ to $3.91 \times 10^{ - 5}$ per base per day. This rate, estimated from comprehensive sequence data obtained by single genome amplification and Sanger sequencing, is comparable to previous estimates of the intra-host HIV-1 nucleotide substitution rate, ranging from $1.72 \times 10^{ - 5}$ to $4.32 \times 10^{ - 5}$ per base per day [20–23]. While previous estimates were based on sequence data collected from either chronic infections or post seroconversion, our estimates are made using sequence data collected initially before immune selection and sampled with intervals of days and weeks. In this way, we were able to monitor the first HIV evolution at the onset of immune selection. Understanding the molecular clock in these early phases is necessary to characterize transmission using a single time point sample. The rate of diversification for 12 of the 15 subjects was statistically comparable to the neutral evolution rate, $2.16 \times 10^{ - 5}$ per base per day, which was previously estimated based on HIV-1 single cycle mutation rate and viral generation time [30, 35]. Over 2 years of infection, HIV-1 diversification began to level off quadratically in 4 of the 15 subjects.

We then investigated the spatial distribution of mutations away from the transmitted/founder sequence across the envelope gene. Mutations were concentrated at putative selection sites, while HIV-1 sequence populations temporally diversify in a clock-like manner with rates consistent with the neutral evolution rate. We classified putative selection sites as positions that showed more base substitutions from the founder sequence than statistically expected. Around 60 % of the statistically identified selection sites were found to be in known CD8+ T cell epitopes from the Los Alamos Immune Database (http://www.hiv.lanl.gov). We then quantified the diversity dynamics within the selection sites as compared to those within non-selection regions. On average, among the 15 subjects examined here, these viral escape sites diversify more than 65 times faster than do non-selection sites, indicating that the majority of mutations accumulate in immune selection sites spanning a small fraction of the entire envelope gene sequence.

Our observations emphasize the ability of multiple escape variants to arise from diverse amino acid changes at given selection sites, with ample evidence as presented in Table 2 [24, 30]. There are several different mutational pathways through which HIV can escape from immune pressure. Viral escapes from cytotoxic T cell responses can be mediated by non-synonymous mutations that can directly abrogate peptide-MHC binding [37, 38]. Escape can also occur via impaired recognition of viral peptide-MHC complexes by cytotoxic T cells [39, 40], or mutations that compromise intracellular epitope processing, for instance, by preventing NH_2_-terminal trimming of the epitope [41]. Antibody escape patterns are also heterogeneous; site-directed mutagenesis has identified multiple resistant variants within the viral envelope CD4 binding site [42]. A considerable amount of sequence variation within the D, V1 and V5 loops, and the CD4-binding site of the HIV-1 envelope has been reported within a subject who developed broadly neutralizing antibody responses [27]. These diverse mechanisms for avoiding immune surveillance sustain multiple mutant lineages during HIV-1 escape.

To address how the molecular clock prevails in a dynamic environment that favors various escape mutants, we proposed a mathematical model describing HIV-1 replication and evolution after transmission. Our model approach allowed us to evaluate the deviation from a constant molecular clock under different immune selection scenarios. When a mutant lineage arises from immune pressure, the most dominant factor of deviation from the clock was the timing of the escape mutant’s appearance; the later it appeared, the greater the deviation. Importantly, the clock deviation was inversely proportional to the number of distinct mutants; when more distinct mutant lineages appeared, viral evolution more closely resembled the molecular clock. This reduction in the clock deviation is due to the fact that distinct mutant lineages most likely coalesce at the founder virus, and thus the greater number of different mutant lineages increases the proportion of virus pairs coalescing to the initial transmission event within the total viral population. Therefore, the capacity for HIV-1 to escape in multiple directions maintains the clock-like evolution of the overall HIV-1 intra-host population.

The presence of multiple mutant lineages can be linked to soft selective sweeps that occur when beneficial mutations are supplied at a rate equal to or greater than once per site per generation [43]. A chronically infected individual is expected to have around $10{}^{8}$ productively infected CD4+ T cells [44]. At the peak of viremia during acute infection, there will be an even greater number of productively infected cells, each being produced by one or more reverse transcription events. Thus, with the mutation rate of $\varepsilon = 2.16 \times 10^{ - 5}$ per base per cycle [33, 35], each selection site is likely to have developed mutations desirable for viral escape before the onset of immune responses. The high mutation rate in parallel with the large HIV-1 population size renders the appearance of multiple mutant lineages very probable, which ensures molecular clock persistence under selection. Similarly, in speciation events, genetic polymorphisms are presumably a major source of multiple mutant lineages in light of the much smaller mutation rate, around $10^{ - 8}$ per base per generation [45].

There are several factors preventing complete adherence to clock-like HIV-1 evolution. Recombination can alter coalescing patterns and thereby perturb clock-like diversification [46]. Hypermutation mediated by APOBEC3G/F can cause the rejection of a single rate molecular clock [47]. Virus latency and compartmentalization may result in viral lineages with different number of replication cycles since the founder virus, as compared to other lineages [48]. Linked homogeneous selection sites can also result in departures from the molecular clock; we observed that temporal deviations from the molecular clock were associated with a greater number of homogeneous selection sites. However, we did not observe hard sweep signatures even within subjects with homogeneity because the fraction of heterogeneous selection sites remained substantial, ranging from 38.5 to 75.5 %. Immune selection patterns are characterized predominantly by soft sweeps, in contrast to HIV-1 drug resistance evolution which involves both hard and soft sweeps [43].

We observed subject-to-subject variations in the rate of early HIV diversification. As previously shown, one of the main parameters affecting the neutral evolution rate is the viral generation time [35]. There is considerable difference in the viral generation time, which is estimated from the slope of plasma HIV-1 RNA decline during antiretroviral therapy [34, 49–52]. However, the accuracy of estimates of viral generation time is complicated by a lack of knowledge of the in vivo drug efficacy in the patients under study. In addition to viral generation time, we may examine other individual-level factors that contribute to variability in diversification rates. For instance, while a recent study observed a greater evolution rate in risk groups with a higher proportion of men [36], we did not observe any differences in the evolution rate between males and females.

The present study provides better opportunities for molecular dating of early HIV infections with a single time point sample. Our observation of clock-like evolution under immune selection validates our approach of dating an early HIV infection using a patient’s Hamming distance distribution [30, 35, 53]. Furthermore, the observed variability in the clock rates necessitates expanding the current method to model the population variability, which could allow for greater precision in estimation of time since infection with a single time point sample. Additionally, the model should be applied to long-term infected individuals with precaution by considering the observed quadratic attenuation in the diversity dynamics for a time frame over 2 years. Developing an accurate tool for estimating the timing of infection is required to meet the growing need for defining immune correlates for protection in on-going vaccine and prevention trials [7].

## Conclusions

In this study of HIV-1 intrahost evolution, we demonstrated that a molecular clock can hold even when a gene phylogeny becomes increasingly complex as the population evolves under selection. By tracing the evolution of HIV-1 at the onset of immune selection, we discovered that (1) 12 out of the 15 subjects’ evolution rate conform to the neutral evolution rate during the first 150 days post infection and (2) in contrast to this regular temporal evolution pattern, mutations were highly clustered on selection sites that diversified more than 65 times faster than non-selection sites. Our mathematical model provides a link between clock conservation and multiple modes of HIV escape. HIV escape complexity was shown to ensure a constant clock-like diversification over time within the first 150 days of infection. The indication of a molecular clock functioning under heavy selection may allow us to date an HIV-1 gene population back to its transmission point, thereby providing crucial information for HIV-1 prevention efforts and grounds for genome-based HIV incidence measures [8, 9].

## Methods

### Sources of published sequence data

A total of 1587 HIV-1 full envelope gene sequences were obtained from 15 subjects’ published data in references, [27, 30–32]. All subjects’ first samples were taken during the acute stage of infection; the first sample of subject CH505 was estimated to have been obtained 4 weeks after infection and the first sample of the other 14 subjects was obtained during Fiebig stage I/II. The sequences from subjects CAP045, CH042, CH131, CH159, CH162, CH164, CH185, CH198, CH256 and CH505 were subtype C and the sequences from subjects CH040, CH058, CH077, SUMA0874 and WEAU0578 were subtype B [27, 30–32]. The subjects did not receive antiretroviral therapy during the period that serial samples were taken [27, 30–32]. Serial HIV-1 envelope gene sequences from the other 11 subjects in these references were excluded in our analysis for the following reasons: subject CAP239 had only two time point samples, subject CH607 received ART, subject CAP210 and CH470 showed the signature of more than a single founder variant, and subjects 1051, 1056, 1058, 1059, 6247, CH607, and TT31P were followed for a period of less than 1 month.

### Mixed effect model for HIV-1 diversity dynamics

We used a linear mixed effects model to analyze the diversity increase over time from the first sample among the 15 subjects. Random coefficients were specified to allow for individual subject deviations from population average regression coefficients for linear and quadratic associations of diversity with time. A mixed effects model for HIV-1 intrahost diversity dynamics is written as,

4 $$d_{i} (t) = (a + \eta_{i} )t + (b + \mu_{i} )t^{2} ,$$

where $d_{i} (t)$ is subject *i*’s diversity increase at time $t$, measured in days, from the first sample, $a + \eta_{i}$ is the linear and $b + \mu_{i}$ is the quadratic coefficient of subject $i$. Here, $a$ is the population linear diversification rate and $b$ is the population quadratic rate. Restricted maximum likelihood was implemented in SAS Proc Mixed to estimate and test the population average and random coefficients. Mixed model estimates were used to evaluate individual subject estimates of linear and quadratic diversity rates.

## Additional file

10.1186/s12977-016-0269-6 Mathematical models for HIV evolution under immune selection, Table S1 (The rate of HIV gene sequence diversification over 2 years from the first sample in 15 subjects), and Table S2 (Documented CD8+ T cell epitopes for statistically designated selection sites from 15 subjects).
