# Supplementary material for: Molecular clock of HIV-1 envelope genes under early immune selection
Source: Retrovirology. 2016 Jun 1;13:38. doi: 10.1186/s12977-016-0269-6 (PMC4888660; doi:10.1186/s12977-016-0269-6)
Supplement: Supplementary file 1 — 10.1186/s12977-016-0269-6 Mathematical models for HIV evolution under immune selection, Table S1 (The rate of HIV gene sequence diversification over 2 years from the first sample in 15 subjects), and Table S2 (Documented CD8+ T cell epitopes for statistically designated selection sites from 15 subjects). [file 12977_2016_269_MOESM1_ESM.docx]

Supplemental Materials

Mathematical models for HIV evolution under immune selection

**One-mutant model: Numbers of wild-type infected cells and mutant cells** As illustrated in Figure 4A, the founder lineage initially replicates in the absence of immune selection, producing $R_{0}$ secondary infected cells from a single infected cell. Each replication cycle involves HIV reverse transcriptase-mediated base errors with the rate, $\varepsilon$, which are the source of viral gene diversification as well as viral escapes from immune surveillance. At the onset of selection at generation $g_{s1}$, a single infected cell harboring an escape virus is assumed to arise at a random location in the tree and begin producing $R_{1}$ daughter cells while the replicative capacity of the wild-type infected cells is compromised, producing only $sR_{0}$ daughters on average with $s<1$. The selection coefficient of the wild-type virus relative to the escape mutant after the onset of selection is $S=1-(sR_{0}/R_{1}).$ When the viral set point is reached the reproductive ratio of the mutant-type infected cell becomes equal to 1 (see Figure 4B), resulting in $N_{m}(g)=R_{1}^{g-g_{s1}}$mutant-type infected cells for $g_{s1}\leq g<g_{s}$ and $N_{m}(g)=R_{1}^{g_{s}-g_{s1}}$ mutant-type infected cells for $g\geq g_{s}$. The infected cell population will also include $N_{w}(g)=(R_{0}^{g_{s1}}-1)(sR_{0})^{g-g_{s1}}$ wild-type infected cells, yielding a total number of infected cells $N(g)=N_{w}(g)+N_{m}(g)$. Note that with a single mutant infected cell, the wild-type infected cell level at generation $g_{s1}$ is $R_{0}^{g_{s1}}-1$. Summarizing,

$N_{w}(g)=\left\{\begin{matrix} R_{0}^{g}\text{ for }0\leq g<g_{s1} \\ (R_{0}^{g_{s1}}-1)(sR_{0})^{g-g_{s1}}\text{ for }g\geq g_{s1} \end{matrix}\right.$ Eq. (S1)

$N_{m}(g)=\left\{\begin{matrix} \text{0 for }0\leq g<g_{s1} \\ R_{1}^{g-g_{s1}}\text{ for }g_{s1}\leq g<g_{s} \\ R_{1}^{g_{s}-g_{s1}}\text{ for }g\geq g_{s} \end{matrix}\right.$ Eq. (S2)

**One-mutant model: Coalescent probability distribution for wild-type viruses** The coalescent probability distribution for a pair of wild-type viruses before the onset of selection follows that of the neutral evolution model. To coalesce at generation $n$, they must be descended from two different $n+1$- generation offspring, yielding a total of $R_{0}^{n}{}_{R_{0}}C_{2}$ $n+1$-generation ancestor set candidates. Since the number of each ancestor’s wild-type descendants at generation $g$ is $R_{0}^{g-n-1}$, the probability of a wild-wild virus pair coalescing at generation $n$ is given by

$\begin{matrix} P_{g<g_{s1}}(MRCA(s_{1},s_{2})=n|s_{1}\in w,s_{2}\in w)=P_{g}^{\text{neutral}}(MRCA(s_{1},s_{2})=n) \\ =\frac{1}{{}_{N_{w}(g)}C{}_{2}}R_{0}^{n}{}_{R_{0}}C_{2}(R_{0}^{g-n-1})^{2}=R_{0}^{-n}\frac{R_{0}^{g-1}(R_{0}-1)}{(R_{0}^{g}-1)}. \end{matrix}$ Eq. (S3)

We first consider that a wild-type infected cell produces more than one descendant under selection, $sR_{0}>1$. The tree topology is modified by immune selection from generation $g_{s1}$ and for two wild-type viruses to coalesce at generation $n$ before the onset of selection ($n<g_{s1}$), they must be descended from two different $n+1$-generation offspring, yielding a total of $R_{0}^{n}{}_{R_{0}}C_{2}$ $n+1$-generation ancestor set candidates. The number of wild-type descendants from each ancestor depends on whether the mutant lineage originates from that ancestor or not. When $q_{1}$ mutant lineages descend from the first wild-type virus’ $n+1$-generation ancestor, the number of wild-type offspring at generation $g$ becomes $\left(R_{0}^{g_{s1}-(n+1)}-q_{1}\right)(sR_{0}\text{)}^{g-g_{s1}}$. When $q_{2}$ mutant lineages descend from the second wild-type virus’ $n+1$-generation ancestor, the number of wild-type descendants becomes $\left(R_{0}^{g_{s1}-(n+1)}-q_{2}\right)(sR_{0}\text{)}^{g-g_{s1}}$. Therefore, the probability of a wild-wild virus pair coalescing at generation $n$ before the onset of selection is given by,

$$\begin{matrix} P_{g\geq g_{sj}}(MRCA(s_{1},s_{2})=n|s_{1}\in w,s_{2}\in w) \\ = \frac{1}{{}_{N_{w}(g)}C_{2}}\sum_{q_{1}=0}^{1} \sum_{q_{2}=0}^{1-q_{1}} P_{w}(q_{1},q_{2};n)R_{0}^{n}{}_{R_{0}}C_{2}\left(R_{0}^{g_{s1}-(n+1)}-q_{1}\right)\left(R_{0}^{g_{s1}-(n+1)}-q_{2}\right)(sR_{0}\text{)}^{2(g-g_{s1})}\text{ for 0}\leq n<g_{s1} \end{matrix}$$

Eq. (S4)

where

$P_{w}(q_{1},q_{2};n)=\frac{{}_{R_{0}^{g_{s1-(n+1)}}}C_{q_{1}}\times{}_{R_{0}^{g_{s1-(n+1)}}}C_{q_{2}}\times{}_{[(R_{0}^{_{n+1}}-2)\times R_{0}^{g_{s1-(n+1)}}]}C_{1-(q_{1}+q_{2})}}{R_{0}^{g_{s1}}},$ Eq. (S5)

is the probability that $q_{1}$ mutant lineages descend from one wild-type virus’ $n+1$-generation ancestor and $q_{2}$ mutant lineages descend from the other wild-type virus’ $n+1$-generation ancestor. Note that $q_{1}$ is either 0 or 1 and $q_{2}$ is either 0 or $1-q_{1}$.

We rearrange Eq. (S5) as follows,

$P_{w}(q_{1},q_{2};n)=\frac{(R_{0}^{g_{s1}}-2)}{R_{0}^{g_{s1}}}\frac{\left(R_{0}^{g_{s1}-(n+1)}\right)^{2}}{\left(R_{0}^{g_{s1}-(n+1)}-q_{1}\right)\left(R_{0}^{g_{s1}-(n+1)}-q_{2}\right)}P_{w}^{'}(q_{1},q_{2};n)$ Eq. (S6)

with

$P_{w}^{'}(q_{1},q_{2};n)=\frac{{}_{[R_{0}^{g_{s1-(n+1)}}-1]}C_{q_{1}}\times{}_{[R_{0}^{g_{s1-(n+1)}}-1]}C_{q_{2}}\times{}_{[(R_{0}^{_{n+1}}-2)\times R_{0}^{g_{s1-(n+1)}}]}C_{1-(q_{1}+q_{2})}}{(R_{0}^{g_{s1}}-2)}.$ Eq. (S7)

Since $\sum_{q_{1}=0}^{1} \sum_{q_{2}=0}^{1-q_{1}} P_{w}^{'}(q_{1},q_{2};n)=1$, Eq. (S4) is simplified as ,

$\begin{matrix} P_{g\geq g_{s1}}(MRCA(s_{1},s_{2})=n|s_{1}\in w,s_{2}\in w) \\ = R_{0}^{-n}\frac{R_{0}^{g_{s1}-1}(R_{0}-1)(R_{0}^{g_{s1}}-2)}{[R_{0}^{g_{s1}}-1-(sR_{0})^{g_{s1}-g}][R_{0}^{g_{s1}}-1]}\text{ for 0}\leq n<g_{s1} \end{matrix}$ Eq. (S8)

Similarly, the probability of two wild-type viruses being coalescent after the onset of selection is calculated as

$\begin{matrix} P_{g\geq g_{s1}}(MRCA(s_{1},s_{2})=n|s_{1}\in w,s_{2}\in w) \\ \text{ =}\frac{1}{{}_{N_{w}(g)}C_{2}}N_{w}(n){}_{sR_{0}}C_{2} (sR_{0})^{2(g-n-1)}\text{=} (sR_{0})^{-n}\frac{(sR_{0})^{g-1}(sR_{0}-1)}{[(R_{0}^{g_{s1}}-1)(sR_{0})^{g-g_{s1}}-1]}\text{ } \text{for g}_{s1}\leq n<g\text{ } \end{matrix}$ Eq. (S9)

By collecting Eq. (S8) and Eq. (S9), the probability of a wild-type virus pair coalescing at generation $n$ when $sR_{0}>1$ is given by

$\begin{matrix} P_{g\geq g_{s1}}(MRCA(s_{1},s_{2})=n|s_{1}\in w,s_{2}\in w) \\ = \left\{\begin{matrix} R_{0}^{-n}\frac{R_{0}^{g_{s1}-1}(R_{0}-1)(R_{0}^{g_{s1}}-2)}{[R_{0}^{g_{s1}}-1-(sR_{0})^{g_{s1}-g}][R_{0}^{g_{s1}}-1]} \text{for 0}\leq n<g_{s1} \\ (sR_{0})^{-n}\frac{(sR_{0})^{g-1}(sR_{0}-1)}{[(R_{0}^{g_{s1}}-1)(sR_{0})^{g-g_{s1}}-1]}\text{ } \text{for }g_{s1}\leq n<g\text{ .} \end{matrix}\right.\text{ } \end{matrix}$ Eq. (S10)

When the replicative capacity of the wild-type infected cells is significantly compromised by immune selection ($sR_{0}\leq1$), the coalescent probability of a wild-type virus pair corresponds to that of the pair’s two ancestors at generation $g_{s1}$ because they cannot coalesce after generation $g_{s1}$. Then the coalescent probability is written as,

$\begin{matrix} P_{g\geq g_{sj}}(MRCA(s_{1},s_{2})=n|s_{1}\in w,s_{2}\in w) \\ = \frac{1}{{}_{(R_{0}^{g_{s1}}-1)}C_{2}}\sum_{q_{1}=0}^{1} \sum_{q_{2}=0}^{1-q_{1}} P_{w}(q_{1},q_{2};n)R_{0}^{n}{}_{R_{0}}C_{2}\left(R_{0}^{g_{s1}-(n+1)}-q_{1}\right)\left(R_{0}^{g_{s1}-(n+1)}-q_{2}\right)\text{ for 0}\leq n<g_{s1} \end{matrix}$ Eq. (S11)

where

$P_{w}(q_{1},q_{2};n)=\frac{{}_{R_{0}^{g_{s1-(n+1)}}}C_{q_{1}}\times{}_{R_{0}^{g_{s1-(n+1)}}}C_{q_{2}}\times{}_{[(R_{0}^{_{n+1}}-2)\times R_{0}^{g_{s1-(n+1)}}]}C_{1-(q_{1}+q_{2})}}{R_{0}^{g_{s1}}},$ Eq. (S12)

is the probability that $q_{1}$ mutant lineages descend from one wild-type virus’ $n+1$-generation ancestor and $q_{2}$ mutant lineages descend from the other wild-type virus’ $n+1$-generation ancestor. Note that $q_{1}$ is either 0 or 1 and $q_{2}$ is either 0 or $1-q_{1}$. By rearranging Eq. (S11) and Eq. (S12), the probability of a wild-type virus pair coalescent at generation $n$ when $sR_{0}\leq1$ is given by

$\begin{matrix} P_{g\geq g_{s1}}(MRCA(s_{1},s_{2})=n|s_{1}\in w,s_{2}\in w) \\ = \left\{\begin{matrix} R_{0}^{-n}\frac{R_{0}^{g_{s1}-1}(R_{0}-1)}{(R_{0}^{g_{s1}}-1)} \text{for 0}\leq n<g_{s1} \\ 0 \text{for }g_{s1}\leq n<g\text{ .} \end{matrix}\right.\text{ } \end{matrix}$ Eq. (S13)

From Eq. (S10) and Eq. (S13), the probability of a wild-type virus pair coalescing at generation $n$ is written as,

$$\begin{matrix} P_{g\geq g_{s1}}(MRCA(s_{1},s_{2})=n|s_{1}\in w,s_{2}\in w) \\ = \left\{\begin{matrix} R_{0}^{-n}\frac{R_{0}^{g_{s1}-1}(R_{0}-1)(R_{0}^{g_{s1}}-2)}{[R_{0}^{g_{s1}}-1-(sR_{0})^{g_{s1}-g}][R_{0}^{g_{s1}}-1]} I(sR_{0}-1) +R_{0}^{-n}\frac{R_{0}^{g_{s1}-1}(R_{0}-1)}{(R_{0}^{g_{s1}}-1)} I(1-sR_{0}) \text{for 0}\leq n<g_{s1} \\ (sR_{0})^{-n}\frac{(sR_{0})^{g-1}(sR_{0}-1)}{[(R_{0}^{g_{s1}}-1)(sR_{0})^{g-g_{s1}}-1]}\text{ }I(sR_{0}-1) \text{for }g_{s1}\leq n<g\text{ ,} \end{matrix}\right.\text{ } \end{matrix}$$

Eq. (S14)

where the indicator function $I(sR_{0}-1)=1$ when $sR_{0}>1$ and $I(sR_{0}-1)=0$otherwise. We can see that this coalesce probability is nearly identical with that of the neutral evolution model in Eq. (S3), which we numerically confirmed. The wild type population, sampled after the onset of selection, most likely coalesces at the founder virus.

**One-mutant model: Coalescent probability distribution for mutant-type viruses** We consider mutant-type virus pairs, from the model assumption that the first mutant-type virus arises at generation $g_{s1}$, we have no mutant-type viruses before then,

$P_{g\leq g_{s1}}(MRCA(s_{1},s_{2})=n|s_{1}\in m,s_{2}\in m)=0.$ Eq. (S15)

The mutant population’s genealogy follows a neutral evolution scenario wherein all mutant descendants originate from a single ancestor at generation $g_{s1}$ with the reproductive ratio, $R_{1}$. Since $R_{1}>1$, the coalescence probabilities of mutant-type infected cells at generation $g$ are dominated by $n=g_{s1}$ as follows:

$\begin{matrix} P_{g_{s1}<g\leq g_{s}}(MRCA(s_{1},s_{2})=n|s_{1}\in m,s_{2}\in m) \\ =\left\{\begin{matrix} 0 \text{for 0}\leq n<g_{s1} \\ R_{1}^{-n}\frac{R_{1}^{g-1}(R_{1}-1)}{R_{1}^{g-g_{s1}}-1} \text{for }g_{s1}\leq n<g \end{matrix}\right. \end{matrix}$ Eq. (S16)

As the reproductive ratio of each mutant-type infected cell is 1 at viral set point ($g>g_{s}$), the probability of coalescing at generations greater than $g_{s}$ becomes zero. In addition, the coalescence probability of a mutant pair becomes that of its two ancestors at generation $g_{s}$,

$\begin{matrix} P_{g>g_{s}}(MRCA(s_{1},s_{2})=n|s_{1}\in m,s_{2}\in m) \\ =\left\{\begin{matrix} 0 \text{for 0}\leq n<g_{s1} \\ \begin{matrix} R_{1}^{-n}\frac{R_{1}^{g_{s}-1}(R_{1}-1)}{R_{1}^{g_{s}-g_{s1}}-1} \text{for }g_{s1}\leq n<g_{s} \\ \text{ 0 for }g_{s}\leq n<g\text{ .} \end{matrix} \end{matrix}\right. \end{matrix}$ Eq. (S17)

As expected, the mutant-type population most likely coalesces at generation$g_{s1}$, the onset of selection and the probability for coalescing at generations greater than $g_{s1}$ decreases as $n$ increases.

**One-mutant model: Coalescent probability distribution for wild-mutant pairs** For a pair of wild-mutant viruses to have a MRCA at generation $n$, the wild-type virus should not be chosen from the offspring of the mutant lineage’s ancestor at generation $n+1$, but from the descendants of the other $R_{0}-1$ cells at generation $n+1$. The total number of those descendants is $(R_{0}-1)\times R_{0}^{g_{s1}-(n+1)}(sR_{0})^{g-g_{s1}}$. Dividing by the number of the wild-type infected cells at generation $g$, $N_{w}(g)$, the coalescent distribution of a pair of the wild-type and mutant-type viruses is given by

$P_{g<g_{s1}}(MRCA(s_{1},s_{2})=n|s_{1}\in w,s_{2}\in m)=0$ Eq. (S18)

$\begin{matrix} P_{g\geq g_{s1}}(MRCA(s_{1},s_{2})=n|s_{1}\in w,s_{2}\in m) \\ \text{ =}\left\{\begin{matrix} R_{0}^{-n}\frac{R_{0}^{g_{s1}-1}(R_{0}-1)}{(R_{0}^{g_{s1}}-1)} \text{for 0}\leq n<g_{s1} \\ \text{ 0 for }g_{s1}\leq n<g\text{ .} \end{matrix}\right. \end{matrix}$ Eq. (S19)

An alternative approach for evaluating coalescence probability of a wild-mutant pair is considering its two ancestors at generation $g_{s1}$. Because the genealogy of the mutant lineage segregates from that of the wild-type lineage from generation $g_{s1}$, the coalescence behavior of a wild-mutant pair can be tracked by that of the pair’s ancestors at generation $g_{s1}$. Therefore, the coalescent distribution of a wild-mutant pair can be written as,

$\begin{matrix} P_{g\geq g_{s1}}(MRCA(s_{1},s_{2})=n|s_{1}\in w,s_{2}\in m) \\ = \left\{\begin{matrix} \frac{R_{0}^{n}{}_{R_{0}}C_{2}\text{ }\left(R_{0}^{g_{s1}-n-1}\right)^{2}}{{}_{R_{0}^{g_{s1}}}C_{2}}=R_{0}^{-n}\frac{R_{0}^{g_{s1}-1}(R_{0}-1)}{R_{0}^{g_{s1}}-1} \text{for 0}\leq n<g_{s1} \\ 0 \text{for }g_{s1}\leq n<g\text{ , } \end{matrix}\right. \end{matrix}$ Eq. (S20)

which is exactly the same as Eq. (S19). The coalescence probability of a wild-type and mutant-type virus pair peaks at the transmission point as does that of a wild-type virus pair.

**One-mutant model: Coalescent probability distribution for any pair of viruses** To obtain the coalescence probability for any random pair of viruses in the population, we take the weighted average of the probability of coalescence for each type of pair,$\begin{matrix} P_{g}(MRCA(s_{1},s_{2})=n) \\ =\frac{1}{{}_{N_{m}(g)+N_{w}(g)}C_{2}}\left\{{}_{N_{w}(g)}C_{2}\text{ }P_{g}(MRCA(s_{1},s_{2})=n|s_{1}\in w,s_{2}\in w)\right. \\ \text{ }+N_{w}(g)N_{m}\text{(g) }P_{g}(MRCA(s_{1},s_{2})=n|s_{1}\in w,s_{2}\in m)\left.\text{+}{}_{N_{m}(g)}C_{2}\text{ }P_{g}(MRCA(s_{1},s_{2})=n|s_{1}\in m,s_{2}\in m)\right\} \end{matrix}$

Eq. (S21)

Illustrating a coalescence genealogy permits us to systematically illustrate the degree of gene diversification of an HIV population. The Hamming distance (HD) of a pair of virus sequences is the number of nucleotide differences between them. The HD distribution of a population of virus sequence pairs can be expressed as a mixture of Poisson distributions with means related to the number of generations elapsed for random mutations to accumulate from the MRCA of each sequence pair. When a virus pair sampled at generation $g$ coalesces at generation $n$, the average HD is given by $\lambda_{g-n}=2\varepsilon(g-n)N_{B}$ , and the probability of an HD of $d$ comes from the Poisson distribution, $\lambda_{g-n}^{d}e^{-\lambda_{g-n}}/d!$ ([1](#_ENREF_1)). By collecting the sequence pairs coalescing from generation 0 to $g-1$, the probability of an HD of $d$ for any virus pairs sampled at generation $g$ becomes,

$P_{g}(HD=d)=\sum_{n=0}^{g-1} \frac{\lambda_{g-n}^{d}e^{-\lambda_{g-n}}}{d!}P_{g}(MRCA(s_{1},s_{2})=n)$ Eq. (S22)

**One-mutant model: deviation from the molecular clock** The deviation of this model from the molecular clock can be quantified as the decrease in the sequence diversity of the sample relative to the reference diversity under neutral evolution,

$\Delta_{1}=\frac{1}{N_{B}}\sum_{n=0}^{g-1} \lambda_{g-n}P_{g}^{\text{neutral}}(MRCA(s_{1},s_{2})=n)-\frac{1}{N_{B}}\sum_{n=0}^{g-1} \lambda_{g-n}\text{ }P_{g}(MRCA(s_{1},s_{2})=n)$ Eq. (S23)

When the mutant lineage is prevalent, the coalescence probability in Eq. (S21) is approximated as,

$P_{g}(MRCA(s_{1},s_{2})=n)\simeq P_{g}(MRCA(s_{1},s_{2})=n|s_{1}\in m,s_{2}\in m)\text{ },$ Eq. (S24)

and thus the clock deviation in Eq. (S23) is approximated as,

$$\begin{matrix} \Delta_{1}\simeq\frac{1}{N_{B}}\sum_{n=0}^{g-1} \lambda_{g-n}P_{g}^{\text{neutral}}(MRCA(s_{1},s_{2})=n)-\frac{1}{N_{B}}\sum_{n=0}^{g-1} \lambda_{g-n}\text{ }P_{g}(MRCA(s_{1},s_{2})=n|s_{1}\in m,s_{2}\in m) \\ \text{ =2}\varepsilon\left\{\sum_{n=g_{s1}}^{g_{s}-1} nR_{1}^{-n}\frac{R_{1}^{g_{s}-1}(R_{1}-1)}{R_{1}^{g_{s}-g_{s1}}-1}-\sum_{n=0}^{g-1} nR_{0}^{-n}\frac{R_{0}^{g-1}(R_{0}-1)}{R_{0}^{g}-1}\right\} \\ \text{ =2}\varepsilon\left\{\frac{R_{1}^{g_{s}-g_{s1}}}{(R_{1}^{g_{s}-g_{s1}}-1)}g_{s1}-\frac{(g_{s}-1)}{(R_{1}^{g_{s}-g_{s1}}-1)}+\frac{R_{1}^{g_{s}-g_{s1}}}{(R_{1}^{g_{s}-g_{s1}}-1)}\frac{1}{(R_{1}-1)}-\frac{1}{(R_{1}^{g_{s}-g_{s1}}-1)}\frac{R_{1}}{(R_{1}-1)}\right. \\ \text{ }\left.-\frac{R_{0}^{g}}{(R_{0}^{g}-1)}\frac{1}{(R_{0}-1)}+\frac{g}{(R_{0}^{g}-1)}+\frac{1}{(R_{0}^{g}-1)}\frac{1}{(R_{0}-1)}\right\}\text{ } \\ \text{ }\simeq2\varepsilon\left\{g_{s1}+\frac{1}{R_{1}-1}-\frac{1}{R_{0}-1}\right\}. \end{matrix}$$

Eq. (S25)

***N*-mutant model** We next generalize the model to the cases where *N* mutant virus lineages arise at random in the phylogeny. The *k*-th type escape virus emerges at generation $g_{sk}$ such that $g_{sk}\geq g_{s(k-1)}$, with its own reproductive ratio $R_{k}$. At generation $g$ before viral set point the number of *k*-th escape viruses is $N_{mk}(g)=R_{k}^{g-g_{sk}}$. At viral set point, the reproductive ratio of all the mutant-type infected cells becomes equal to 1, giving $N_{mk}(g)=R_{k}^{g_{s}-g_{sk}}$ for $g\geq g_{s}$.

The coalescing probability for virus pairs within *k*-th mutant lineage is given by,

$P_{g\leq g_{sk}}(MRCA(s_{1},s_{2})=n|s_{1}\in m_{k},s_{2}\in m_{k})=0\text{ ,}$ Eq. (S26)

$\begin{matrix} P_{g_{sk}<g\leq g_{s}}(MRCA(s_{1},s_{2})=n|s_{1}\in m_{k},s_{2}\in m_{k}) \\ =\left\{\begin{matrix} 0 \text{for 0}\leq n<g_{sk} \\ R_{k}^{-n}\frac{R_{k}^{g-1}(R_{k}-1)}{R_{k}^{g-g_{sk}}-1} \text{for }g_{sk}\leq n<g\text{ ,} \end{matrix}\right. \end{matrix}$ Eq. (S27)

$\begin{matrix} P_{g>g_{s}}(MRCA(s_{1},s_{2})=n|s_{1}\in m_{k},s_{2}\in m_{k}) \\ =\left\{\begin{matrix} 0 \text{for 0}\leq n<g_{sk} \\ \begin{matrix} R_{k}^{-n}\frac{R_{k}^{g_{s}-1}(R_{k}-1)}{R_{k}^{g_{s}-gk}-1} \text{for }g_{sk}\leq n<g_{s} \\ \text{ 0 for }g_{s}\leq n<g\text{ .} \end{matrix} \end{matrix}\right. \end{matrix}$ Eq. (S28)

The coalescent probability of virus pairs from two different mutant lineages, $i$ and $j$, corresponds to that of the pair’s two wild-type ancestors at generation $g_{si}$. The probability of wild-type infected cells having a MRCA after generation $g_{s1}$ is zero when the replicative capacity of the wild-type infected cells is significantly reduced, $sR_{0}<1$. Thus, the coalescent probability of two $g_{si}$-generation ancestors becomes equal to that of two $g_{s1}$-generation ancestors of *i*-th and *j*-th mutant-type viruses. To coalesce to one of $R_{0}^{n}$ available MRCA candidates, *i*-th and *j*-th mutant lineages’ $g_{s1}$-generation ancestors should originate from two different $n+1$-generation offspring. When $q_{1}$ mutant lineages out of other $N-2$ mutant lineages descend from the *i*-th mutant lineages’ $n+1$-generation ancestor, the number of choices for *i*-th mutant lineage’s $g_{s1}$-generation ancestor is $R_{0}^{g_{s1}-(n+1)}-q_{1}$. Likewise the number of choices for *j*-th mutant lineage’s $g_{s1}$-generation ancestor is $R_{0}^{g_{s1}-(n+1)}-q_{2}$ when $q_{2}$ other mutant lineages descend from the *j*-th mutant lineages’ $n+1$-generation ancestor. Thus, the probability of a virus pair from two different mutant lineages coalescing at generation $n$ is given by,

$$P_{g<g_{sj}}(MRCA(s_{1},s_{2})=n|s_{1}\in m_{i},s_{2}\in m_{j})=0\text{ ,}$$

$\begin{matrix} P_{g\geq g_{sj}}(MRCA(s_{1},s_{2})=n|s_{1}\in m_{i},s_{2}\in m_{j}) \\ = \left\{\begin{matrix} \frac{1}{{}_{[R_{0}^{g_{s1}}-(N-2)]}C_{2}}\sum_{q_{1}=0}^{N-2} \sum_{q_{2}=0}^{N-2-q_{1}} P_{m}(q_{1},q_{2};n)R_{0}^{n}{}_{R_{0}}C_{2}\left(R_{0}^{g_{s1}-(n+1)}-q_{1}\right)\left(R_{0}^{g_{s1}-(n+1)}-q_{2}\right) \text{for 0}\leq n<g_{s1} \\ 0 \text{for }g_{s1}\leq\text{ }n<g\text{,} \end{matrix}\right. \end{matrix}$ Eq. (S29)

where

$P_{m}(q_{1},q_{2};n)=\frac{{}_{R_{0}^{g_{s1-(n+1)}}}C_{q_{1}}\times{}_{R_{0}^{g_{s1-(n+1)}}}C_{q_{2}}\times{}_{[(R_{0}^{_{n+1}}-2)\times R_{0}^{g_{s1-(n+1)}}]}C_{N-2-(q_{1}+q_{2})}}{{}_{R_{0}^{g_{s1}}}C_{N-2}},$ Eq. (S30)

is the probability that $q_{1}$ mutant lineages descend from the *i*-th mutant lineage’s $n+1$-generation ancestor and $q_{2}$ mutant lineages descend from the *j*-th mutant lineage’s $n+1$-generation ancestor. This probability is evaluated by counting the number of ways to randomly choose the $N-2$ mutant lineages’ $g_{s1}$-generation ancestors out of $R_{0}^{g_{s1}}$ cells at generation $g_{s1}$, ${}_{R_{0}^{g_{s1}}}C_{N-2}$, and the number of ways to choose $q_{1}$, $q_{2}$, and $N-2-q_{1}-q_{2}$mutant lineages’ $g_{s1}$-generation ancestors out of offspring of the *i*-th mutant lineage’s $n+1$-generation ancestor, offspring of the *j*-th mutant lineage’s $n+1$-generation ancestor, and offspring of the other $R_{0}^{n+1}-2$ ancestors at generation $n+1$, respectively. Note that $q_{1}$ ranges from 0 to $N-2$ and $q_{2}$ from 0 to $N-2-q_{1}$. We rewrite Eq. (S30) as,

$\begin{matrix} P_{m}(q_{1},q_{2};n)=\frac{{}_{[R_{0}^{g_{s1}}-(N-2)]}C_{2}}{{}_{R_{0}^{g_{s1}}}C_{2}}\frac{\left(R_{0}^{g_{s1}-(n+1)}\right)^{2}}{\left(R_{0}^{g_{s1}-(n+1)}-q_{1}\right)\left(R_{0}^{g_{s1}-(n+1)}-q_{2}\right)}\frac{{}_{[R_{0}^{g_{s1-(n+1)}}-1]}C_{q_{1}}\times{}_{[R_{0}^{g_{s1-(n+1)}}-1]}C_{q_{2}}\times{}_{[(R_{0}^{_{n+1}}-2)\times R_{0}^{g_{s1-(n+1)}}]}C_{N-2-(q_{1}+q_{2})}}{{}_{R_{0}^{g_{s1}}-2}C_{N-2}} \\ \text{ =}\frac{{}_{[R_{0}^{g_{s1}}-(N-2)]}C_{2}}{{}_{R_{0}^{g_{s1}}}C_{2}}\frac{\left(R_{0}^{g_{s1}-(n+1)}\right)^{2}}{\left(R_{0}^{g_{s1}-(n+1)}-q_{1}\right)\left(R_{0}^{g_{s1}-(n+1)}-q_{2}\right)}P_{m}^{'}(q_{1},q_{2};n), \end{matrix}$ Eq. (S31)

with

$$P_{m}^{'}(q_{1},q_{2};n)=\frac{{}_{[R_{0}^{g_{s1-(n+1)}}-1]}C_{q_{1}}\times{}_{[R_{0}^{g_{s1-(n+1)}}-1]}C_{q_{2}}\times{}_{[(R_{0}^{_{n+1}}-2)\times R_{0}^{g_{s1-(n+1)}}]}C_{N-2-(q_{1}+q_{2})}}{{}_{R_{0}^{g_{s1}}-2}C_{N-2}}.$$

Then, Eq. (S29) becomes

$\begin{matrix} P_{g\geq g_{sj}}(MRCA(s_{1},s_{2})=n|s_{1}\in m_{i},s_{2}\in m_{j}) \\ = \left\{\begin{matrix} \frac{1}{{}_{R_{0}^{g_{s1}}}C_{2}}\sum_{q_{1}=0}^{N-2} \sum_{q_{2}=0}^{N-2-q_{1}} P_{m}^{'}(q_{1},q_{2};n)R_{0}^{n}{}_{R_{0}}C_{2}\left(R_{0}^{g_{s1}-(n+1)}\right)^{2} \text{for 0}\leq n<g_{s1} \\ 0 \text{for }g_{s1}\leq\text{ }n<g\text{,} \end{matrix}\right. \end{matrix}$ Eq. (S32)

Since $\sum_{q_{1}=0}^{N-2} \sum_{q_{2}=0}^{N-2-q_{1}} P_{m}^{'}(q_{1},q_{2};n)=1$, the probability of a virus pair from two different mutant lineages is simplified as,

$\begin{matrix} P_{g\geq g_{sj}}(MRCA(s_{1},s_{2})=n|s_{1}\in m_{i},s_{2}\in m_{j}) \\ \text{ } = \left\{\begin{matrix} R_{0}^{-n}\frac{R_{0}^{g_{s1}-1}(R_{0}-1)}{(R_{0}^{g_{s1}}-1)} \text{for 0}\leq n<g_{s1} \\ 0 \text{for }g_{s1}\leq\text{ }n<g\text{ . } \end{matrix}\right. \end{matrix}$ Eq. (S33)

The MRCA of the mutant viruses originating from different lineages is most likely the founder virus and the coalescent profile is nearly identical with that of the neutral model.

The coalescence probability of two wild-type viruses, when $sR_{0}<1$, can be obtained following the same procedure above by considering two $g_{s1}$-generation ancestors of the wild-type virus pair,

$$P_{g<g_{s1}}(MRCA(s_{1},s_{2})=n|s_{1}\in w,s_{2}\in w)=P_{g}^{\text{neutral}}\text{(}MRCA(s_{1},s_{2}\text{)}=n)\text{ ,}$$

$\begin{matrix} P_{g\geq g_{s1}}(MRCA(s_{1},s_{2})=n|s_{1}\in w,s_{2}\in w) \\ \text{ }= \left\{\begin{matrix} \frac{1}{{}_{(R_{0}^{g_{s1}}-N)}C_{2}}\sum_{q_{1}=0}^{N} \sum_{q_{2}=0}^{N-q_{1}} P_{w}(q_{1},q_{2};n)R_{0}^{n}{}_{R_{0}}C_{2}\left(R_{0}^{g_{s1}-(n+1)}-q_{1}\right)\left(R_{0}^{g_{s1}-(n+1)}-q_{2}\right) \text{for 0}\leq n<g_{s1} \\ 0 \text{for }g_{s1}\leq\text{ }n<g\text{ ,} \end{matrix}\right. \end{matrix}$ Eq. (S34)

where

$P_{w}(q_{1},q_{2};n)=\frac{{}_{R_{0}^{g_{s1-(n+1)}}}C_{q_{1}}\times{}_{R_{0}^{g_{s1-(n+1)}}}C_{q_{2}}\times{}_{[(R_{0}^{_{n+1}}-2)\times R_{0}^{g_{s1-(n+1)}}]}C_{N-(q_{1}+q_{2})}}{{}_{R_{0}^{g_{s1}}}C_{N}},$ Eq. (S35)

is the probability that $q_{1}$ mutant lineages descend from the first wild-type virus’ $n+1$-generation ancestor and $q_{2}$ mutant lineages descend from the other wild-type virus’ $n+1$-generation ancestor. By rearranging Eq. (S34) and Eq. (S35), the coalescent probability of a wild-type virus pair is simplified as,

$\begin{matrix} P_{g\geq g_{1}}(MRCA(s_{1},s_{2})=n|s_{1}\in w,s_{2}\in w) \\ \text{ } = \left\{\begin{matrix} R_{0}^{-n}\frac{R_{0}^{g_{s1}-1}(R_{0}-1)}{(R_{0}^{g_{s1}}-1)} \text{for 0}\leq n<g_{s1} \\ 0 \text{for }g_{s1}\leq\text{ }n<g\text{ . } \end{matrix}\right. \end{matrix}$ Eq. (S36)

The wild-type virus pairs most likely coalesce at the founder virus.

Similarly, the coalescence probability of a pair of wild-mutant viruses is written as,

$$P_{g<g_{si}}(MRCA(s_{1},s_{2})=n|s_{1}\in w,s_{2}\in m_{i})=0\text{ ,}$$

$\begin{matrix} P_{g\geq g_{si}}(MRCA(s_{1},s_{2})=n|s_{1}\in w,s_{2}\in m_{i}) \\ = \left\{\begin{matrix} \frac{1}{{}_{[R_{0}^{g_{s1}}-(N-1)]}C_{2}}\sum_{q_{1}=0}^{N-1} \sum_{q_{2}=0}^{N-1-q_{1}} P_{mw}(q_{1},q_{2};n)R_{0}^{n}{}_{R_{0}}C_{2}\left(R_{0}^{g_{s1}-(n+1)}-q_{1}\right)\left(R_{0}^{g_{s1}-(n+1)}-q_{2}\right) \text{for 0}\leq n<g_{s1} \\ 0 \text{for }g_{s1}\leq\text{ }n<g\text{ ,} \end{matrix}\right. \end{matrix}$ Eq. (S37)

where

$P_{wm}(q_{1},q_{2};n)=\frac{{}_{R_{0}^{g_{s1-(n+1)}}}C_{q_{1}}\times{}_{R_{0}^{g_{s1-(n+1)}}}C_{q_{2}}\times{}_{[(R_{0}^{_{n+1}}-2)\times R_{0}^{g_{s1-(n+1)}}]}C_{N-1-(q_{1}+q_{2})}}{{}_{R_{0}^{g_{s1}}}C_{N-1}},$ Eq. (S38)

is the probability that $q_{1}$ mutant lineages descend from the wild-type virus’ $n+1$-generation ancestor and $q_{2}$ mutant lineages descend from *i*-th mutant lineage’s $n+1$- generation ancestor. By rearranging Eq. (S37) and Eq. (S38), we have

$\begin{matrix} P_{g\geq g_{i}}(MRCA(s_{1},s_{2})=n|s_{1}\in w,s_{2}\in m_{i}) \\ \text{ } = \left\{\begin{matrix} R_{0}^{-n}\frac{R_{0}^{g_{s1}-1}(R_{0}-1)}{(R_{0}^{g_{s1}}-1)} \text{for 0}\leq n<g_{s1} \\ 0 \text{for }g_{s1}\leq\text{ }n<g\text{ . } \end{matrix}\right. \end{matrix}$ Eq. (S39)

The MRCA of pairs of wild-mutant viruses is also most likely the founder virus. From Eqs. (S33), (S36), and (S39), we conclude that the MRCA of any virus pairs except pairs within a single mutant lineage is most likely the founder virus even under the increasingly complex phylogeny with $N$mutant lineages.

In the extreme limit where a single mutant lineage, for instance, the *i*-th mutant-type lineage, becomes dominant due to its high reproductive ratio and/or early appearance after most of the wild-type population is cleared out, the coalescence probability for any random virus pair in the population is approximated as,

$P_{g}(MRCA(s_{1},s_{2})=n)\simeq P_{g}(MRCA(s_{1},s_{2})=n|s_{1}\in m_{i},s_{2}\in m_{i})$, Eq. (S40)

and the deviation from the molecular clock is approximated as

$$\begin{matrix} \Delta_{N}=\frac{1}{N_{B}}\sum_{n=0}^{g-1} \lambda_{g-n}\text{ }P_{g}^{\text{neutral}}(MRCA(s_{1},s_{2})=n)-\frac{1}{N_{B}}\sum_{n=0}^{g-1} \lambda_{g-n}\text{ }P_{g}(MRCA(s_{1},s_{2})=n) \\ \text{ }\simeq\frac{1}{N_{B}}\sum_{n=0}^{g-1} \lambda_{g-n}\text{ }P_{g}^{\text{neutral}}(MRCA(s_{1},s_{2})=n)-\frac{1}{N_{B}}\sum_{n=0}^{g-1} \lambda_{g-n}\text{ }P_{g}(MRCA(s_{1},s_{2})=n|s_{1}\in m_{i},s_{2}\in m_{i}) \\ \text{ = }\Delta_{1} \end{matrix}$$

Eq. (S41)

When one mutant lineage prevails in the viral population, the *N*-mutant model effectively behaves like the one mutant model, resulting in the same amount of the clock deviation. However, early HIV evolution signatures support the coexistence of multiple mutant lineages and thus we consider the other extreme where the population size of each mutant lineage is comparable to one another in the generalized model. Then the coalescence probability is approximated as,

$\begin{matrix} P_{g}(MRCA(s_{1},s_{2})=n) \\ \simeq\frac{1}{{}_{N\times N_{mi}(g)}C_{2}}\left\{N{}_{N_{mi}(g)}C_{2}\text{ }P_{g}(MRCA(s_{1},s_{2})=n|s_{1}\in m_{i},s_{2}\in m_{i})\right. \\ \left.\text{ }+{}_{N}C_{2}\text{ }N_{mi}(g)N_{mj}(g)P_{g}(MRCA(s_{1},s_{2})=n|s_{1}\in m_{i},s_{2}\in m_{j})\right\} \\ \simeq\frac{1}{N}P_{g}(MRCA(s_{1},s_{2})=n|s_{1}\in m_{i},s_{2}\in m_{i})+P_{g}(MRCA(s_{1},s_{2})=n|s_{1}\in m_{i},s_{2}\in m_{j}) \\ \text{ }-\frac{1}{N}P_{g}(MRCA(s_{1},s_{2})=n|s_{1}\in m_{i},s_{2}\in m_{j}). \end{matrix}$ Eq. (S42) In this limit, the deviation from the molecular clock becomes

$\begin{matrix} \Delta_{N}=\frac{1}{N_{B}}\sum_{n=0}^{g-1} \lambda_{g-n}\text{ }P_{g}^{\text{neutral}}(MRCA(s_{1},s_{2})=n)-\frac{1}{N_{B}}\sum_{n=0}^{g-1} \lambda_{g-n}\text{ }P_{g}(MRCA(s_{1},s_{2})=n) \\ \text{ }\simeq\frac{1}{N_{B}}\sum_{n=0}^{g-1} \lambda_{g-n}\text{ }P_{g}^{\text{neutral}}(MRCA(s_{1},s_{2})=n)-\frac{1}{N_{B}}\sum_{n=0}^{g-1} \lambda_{g-n}\text{ }P_{g}(MRCA(s_{1},s_{2})=n|s_{1}\in m_{i},s_{2}\in m_{j}) \\ \text{+}\frac{1}{N}\left[\frac{1}{N_{B}}\sum_{n=0}^{g-1} \lambda_{g-n}\text{ }P_{g}(MRCA(s_{1},s_{2})=n|s_{1}\in m_{i},s_{2}\in m_{j})-\frac{1}{N_{B}}\sum_{n=0}^{g-1} \lambda_{g-n}\text{ }P_{g}(MRCA(s_{1},s_{2})=n|s_{1}\in m_{i},s_{2}\in m_{i})\right]. \end{matrix}$Since $P_{g}(MRCA(s_{1},s_{2})=n|s_{1}\in m_{i},s_{2}\in m_{j})\simeq P_{g}^{\text{neutral}}(MRCA(s_{1},s_{2})=n)\text{ }$from Eq. (S3) and Eq. (S33), we have

$\Delta_{N}\simeq\frac{\Delta_{1}}{N}$, Eq. (S43)

where $\Delta_{1}$ is the clock deviation of the single mutant model as derived in Eq. (S25). The deviation from the molecular clock, measured by the sequence diversity change from the reference clock, decreases as the number of mutant lineages increases. In conclusion, a selection-induced heterogeneous phylogeny can conform to a strict molecular clock when many mutant lineages coexist. This is because different mutant lineages most likely coalesce at the founder virus.

| Subject | Rate of linear diversification  with standard errors  ($\times10^{-5}$ per base  per day) | Rate of quadratic diversification with standard errors  ($\times10^{-5}$ per base  per day^2^) | The mixed effect model p-values  (linear / quadratic) | P-value for departure  from the linear neutral rate* |
| --- | --- | --- | --- | --- |
| CAP045 | 3.24$\pm$0.56 | $-$0.0012 $\pm$0.0012 | 0.0017 / 0.34 | 0.67 |
| CH040 | 1.54 $\pm$0.39 | $-$0.00073 $\pm$0.0010 | 0.0021 / 0.48 | 0.20 |
| CH042 | 1.96 $\pm$0.61 | 0.000087 $\pm$0.00063 | < 0.0001 / 0.89 | 0.78 |
| CH058 | 2.91 $\pm$0.51 | $-$0.0017 $\pm$ 0.0012 | 0.0021 / 0.20 | 0.51 |
| CH077 | 3.91 $\pm$0.42 | $-$0.0026 $\pm$0.00083 | <0.0001 / 0.0076 | 0.11 |
| CH131 | 2.21 $\pm$0.38 | $-$0.0027 $\pm$ 0.00050 | < 0.0001 / < 0.0001 | 0.016 |
| CH159 | 2.57 $\pm$ 0.42 | $-$0.0020 $\pm$0.00068 | < 0.0001 / 0.012 | 0.047 |
| CH162 | 2.46 $\pm$0.53 | $-$0.00094 $\pm$0.0010 | 0.0024 / 0.38 | 0.32 |
| CH164 | 2.26 $\pm$0.64 | 0.00017 $\pm$0.0011 | 0.013 / 0.88 | 0.095 |
| CH185 | 1.86 $\pm$0.57 | $-$0.00042 $\pm$0.0011 | 0.041 / 0.70 | 0.024 |
| CH198 | 3.57 $\pm$0.62 | $-$0.0022 $\pm$0.0013 | < 0.0001 / 0.10 | 0.047 |
| CH256 | 3.58 $\pm$0.58 | $-$0.0013 $\pm$0.00061 | < 0.0001 / 0.055 | 0.23 |
| CH505 | 2.30 $\pm$0.27 | $-$0.0011 $\pm$0.00052 | < 0.0001 / 0.046 | 0.16 |
| SUMA0874 | 2.85 $\pm$0.78 | $-$0.0014 $\pm$0.0014 | 0.012 / 0.33 | 0.77 |
| WEAU0578 | 3.17 $\pm$0.79 | $-$0.0012 $\pm$0.0011 | 0.0006 / 0.31 | 0.89 |
| Population mean | 2.69 $\pm$0.29 | $-$0.00122 $\pm$0.000497 | N/A | N/A |

**Table S1.** The rate of HIV gene sequence diversification over 2 years from the first sample in 15 subjects whose sequence data come from references ([2](#_ENREF_2)), ([3](#_ENREF_3)), ([4](#_ENREF_4)), and ([5](#_ENREF_5)).

*Less than 0.05 implies statistically a significant deviation from the neutral evolution rate.

| **Subject** | **Selection Sites**  **(gp160)** | **CD8+ T cell epitope (gp160)** | **HLA Restriction** |
| --- | --- | --- | --- |
| CAP045 | 51 | VPVWKEATTTL (42-52) | B35 |
|  | 186, 187 | YSENSSEYY (183-191) | A*01 |
|  | 278 | IRSENLTNNAKTIIVHL (272-288) | C*06 |
|  | 334, 339 | SRAKWNNTL (334-342) | B27 |
|  | 360 | QFRNKTIVF (352-361) | Cw*0401 |
|  | 363, 364 | QSSGGDPEIVTHSF (363-376) | B18 |
|  | 752 | RSIRLVSGFL (744-753) | human |
|  | 812 | QELKNSAVSL (805-814) | B*4001 |
| CH040 | 147, 148 | TYNETYNEI (146-154) | human |
|  | 295 | VEINCTR (292-298) | **A2** |
|  | 323, 330 | EIIGDIRQAY (321-330) | A*2501 |
|  | 419 | ITLPCRIKQIINMWQ (414-428) | A*3201 |
|  | 500 | LGVAPTTTKRRWER (494-508) | human |
|  | 555 | LLRAIEAQQHL (555-565) | A11.1 |
|  | 754 | RLVNGSLAL (747-755) | **A2** |
|  | 780 | **LFSYHRLRDLLLIVTRIVE (765-783)** | **A*3101** |
|  | 820 | SLLNATDIAV (813-822) | **A*0201** |
| CH042 | 30 | AAENLWVTVYY (30-40) | B44 |
|  | 32 | AENLWVTVY (31-39) | **B*1801** |
|  | 113 | IISLWDQSL (108-116) | A*0201 |
|  | 145 | RMIMEKGEI* (146-153) | Human |
|  | 170, 171, 173, 175 | **VQKEYAFFYK (169-178)** | **A2** |
|  | 185 | YSENSSEYY (183-191) | A*01 |
|  | 239 | GPCKNVSTVQ (237-346) | B56 |
|  | 305 | CTRPNNNTRK (296-305) | A2, A3 |
|  | 325, 330 | EIIGDIRQAY (321-330) | A*2501 |
|  | 336 | SRAKWNNTL (334-342) | B27 |
|  | 367 | QSSGGDPEIVTHSF (363-376) | **B18** |
|  | 379 | SFNCGGEFF (375-383) | **B15** |
|  | 394 | STWNVNGTW (393-400) | B27 |
|  | 422 | LPCRIKQII (416-424) | B*5101 |
|  | 446 | RCSSNITGLL (444-453) | B56 |
|  | 500 | APTKAKRRV (497-505) | Human |
|  | 612 | TTTVPWNVSW (605-614) | B*5701 |
|  | 640, 641, 654 | **NYTSLIHSLIEESQNQQE (637-654)** | Cw*0702 |
|  | 756 | VTGFLALAW (749-757) | human |
|  | 783 | IVTRIVELL (777-785) | A*6802 |
| CH058 | 586, 588 | **ERYLRDQQL (584-592)** | **B*1402** |
|  | 836 | RVIEVLQRA (828-836) | A*0201 |
|  | 840 | RAYRAILHI (835-843) | B51 |
| CH077 | 5, 10 | RVKEKYQHL (2-10) | B*0801 |
|  | 60 | KAYEKEVHNVW (59-69) | **B*5703** |
|  | 152 | RMIMEKGEI (146-153) | Human |
|  | 168 | IRDKVQKEY (165-173) | B27 |
|  | 187, 188 | YSENSSEYY (183-191) | A*01 |
|  | 210 | VSFEPIPPHYCA (208-219) | **A2** |
|  | 241 | GPCKNVSTVQ (237-246) | B56 |
|  | 295, 297 | VEINCTR (292-298) | **A2** |
|  | 344 | **SGEDWNKTLSHVVDKLRE (334-351)** | A*0201 |
|  | 354 | **QFRNKTIVF (352-361)** | **Cw*0401** |
|  | 386 | KNCGGEFFYCNS (376-387) | **A2** |
|  | 612 | TTTVPWNVSW (605-614) | **B*5701** |
|  | 746 | EQDRGRSIRLVSGFL (739-753) | Human |
|  | 821 | LLDTIAIAV (814-822) | **A*0205** |
|  | 829, 832 | **VAEGTDRVIEELQRAWRA (822-839)** | **A2** |
|  | 843 | IPRRIRQGL (843-851) | A*0201 |
| CH131 | 12 | TQMNWPNLWK (6-12) | A11 |
|  | 57 | TTLFCASDAK (50-59) | A*0301 |
|  | 80 | DPNPQEVVL (78-86) | B*3501 |
|  | 155 | KNCSFNMTT (155-163) | Human |
|  | 166, 171 | IRDKVQKEY (165-173) | B27 |
|  | 190 | YSENSSEYY (183-191) | A*01 |
|  | 197 | YRLINCNTSV (191-200) | A2 |
|  | 209 | KMSFEPIPIH (207-216) | **A29** |
|  | 274 | EVVIRSVNFTDNA (269-281) | Human |
|  | 293, 295 | VEINCTR (292-298) | A2 |
|  | 308 | CTRPNNNTRKSVRIG (296-310) | B*0702 |
|  | 330 | EIIGDIRQAY (321-330) | A*2501 |
|  | 334 | SRAKWNNTL (334-342) | B27 |
|  | 344 | RVLKQVTEK (340-348) | A11 |
|  | 373 | PEIVTHS (369-375) | A2 |
|  | 396 | STWNVNGTW (393-400) | B27 |
|  | 512 | RAVGMGALIFEFL (511-523) | Human |
|  | 717 | **RQGYSPLSFQTLIPNPRG (109-726)** | **Cw*0102** |
|  | 744, 750 | RSIRLVSGFL (744-753) | human |
|  | 843 | IPRRIRQGL (843-851) | A*0201- |
| CH159 | 50, 51, 56 | TTLFCASDAK (50-59) | A*0301 |
|  | 161 | NCSFNISTSI (156-165) | Cw8 |
|  | 166, 168 | IRDKVQKEY (165-173) | B27 |
|  | 190 | YSENSSEYY (183-191) | A*01 |
|  | 193 | YRLINCNTSV (191-200) | A2 |
|  | 254 | RPIVSTQLL (252-260) | B*3501 |
|  | 274 | KIAIRSENISNNA (269-281) | Human |
|  | 295 | SVEINCTRPNNNTRKSI (291-307) | A2 |
|  | 321 | GRAFVTIGK (314-322) | B*2705 |
|  | 328, 329 | EIIGDIRQAY (321-330) | A*2501 |
|  | 334 | SRAKWNNTL (334-342) | B27 |
|  | 344, 347 | RVLKQVTEK (340-348) | A11 |
|  | 369 | QSSGGDPEIVTHSF (363-376) | **B18** |
|  | 417 | ITLPCRIKQIINMWQ (414-428) | A*3201 |
|  | 446 | RCSSNITGLL (444-453) | B56 |
|  | 504 | VKIEPLGVAPTKAKRRVVQR (489-508) | A2 |
|  | 515 | RAVGIGAVFLGFLGAA (511-526) | C*08 |
|  | 580 | QTRVLAIERYL (577-582) | B*5802 |
|  | 644 | **MEWDREINNYTSLIHSLIEESQNQQE (629-654)** | Cw*0702 |
|  | 691 | FIMIVGGLV (685-693) | A*0201 |
|  | 816 | SLLNATDIAV (813-822) | **A68** |
| CH162 | 32 | AENLWVTVY (31-39) | **B*4403** |
|  | 117 | KPCVKLIPL (117-125) | **B*4202** |
|  | 162, 164 | NCSFNISTSI (156-165) | Cw*08 |
|  | 169 | IRDKVQKEY (165-173) | B27 |
|  | 295 | SVEINCTRPNNNTRKSI (291-307) | A2 |
|  | 389 | FYCNTTQLF (383-391) | A*2402 |
|  | 397, 400 | STWNVNGTW (393-400) | B27 |
|  | 493 | YKVVKIEPL (486-494) | Human |
|  | 750 | EQDRGRSIRLVSGFL (739-753) | Human |
|  | 783 | DFILIVARTVELLGH (773-787) | A*6802 |
|  | 804 | LLQYWSQEL (799-807) | A*0201 |
|  | 819 | SLLNATDIAV (813-822) | A*0201 |
| CH164 | 5 | RVMETRRSW (2-10) | B*0801 |
|  | 156 | NCSFNISTSI (156-165) | Cw8 |
|  | 210 | **SFEPIPIHY* (209-217)** | **A*2902** |
|  | 262 | **LNGSLAEE (261-278)** | B7 |
|  | 340 | SRAKWNNTL (334-342) | B27 |
|  | 358, 359, 361 | QFRNKTIVF (352-361) | Cw*0401 |
|  | 399 | STWNVNGTW (393-400) | B27 |
|  | 466, 468 | EVFRPGGGDM (466-475) | A*2601 |
|  | 745 | EQDRGRSIRLVSGFL (739-753) | human |
| CH185 | 153 | TYNETYNEI (146-154) | Human |
|  | 162 | NCSFNISTSI (156-165) | Cw8 |
|  | 187 | YSENSSEYY (183-191) | **A*01** |
|  | 364 | QSSGGDPEIVTHSF* (363-376) | B18 |
|  | 515 | RAVGIGAVFLGFLGAA (511-526) | C*08 |
|  | 704 | VFAVLSIVNR (698-707) | A*3303 |
|  | 762 | LRSLFLFS (760-767) | A*2301 |
|  | 796, 801 | KYCWNLLQY (794-802) | A*3002 |
| CH198 | 7 | RVKEKYQHL (2-10) | **B*0801** |
|  | 51 | TTLFCASDAK (50-59) | **A*0301** |
|  | 148, 149 | RMIMEKGEI (146-153) | human |
|  | 176 | KVQKEYAFF (168-176) | A2 |
|  | 188 | YSENSSEYY (183-191) | A*01 |
|  | 196 | TLTSCNTSV (192-200) | A*0201 |
|  | 324 | EIIGDIRQAY (321-330) | A*2501 |
|  | 338, 341 | SRAKWNNTL (334-343) | B27 |
|  | 345 | RVLKQVTEK (341-349) | A*0201 |
|  | 364, 365 | QSSGGDPEIVTHSF (363-376) | B18 |
|  | 397, 398, 399, 400 | STWNVNGTW (393-400) | B27 |
|  | 449, 450, 451 | RCSSNITGLL (444-453) | B56 |
|  | 469, 470, 471 | EVFRPGGGDM (466-475) | A*2601 |
|  | 536 | TMGAASITL (529-537) | A2 |
|  | 839 | RAYRAILHI (835-843) | B*5101 |
| CH256 | 85 | DPNPQEVVL (78-86) | B*3501 |
|  | 125 | KLTPLCVTL (121-129) | A*0201 |
|  | 146, 153 | RMIMEKGEI (146-153) | Human |
|  | 154 | RMIMEKGEI (146-153) | Human |
|  | 186, 188, 190 | YSENSSEYY (183-191) | A*01 |
|  | 192 | YRLINCNTSV (191-200) | A2 |
|  | 244 | CKNVSTVQC (239-247) | **Cw*0802** |
|  | 259 | RPIVSTQLL (252-260) | B*3501 |
|  | 284 | NAKTIIVHL (280-288) | Cw*0602 |
|  | 334, 338 | SRAKWNNTL (334-342) | B27 |
|  | 343, 347 | RVLKQVTEK (340-348) | A11 |
|  | 365, 366 | QSSGGDPEIVTHSF (363-376) | B18 |
|  | 395 | STWNVNGTW (393-400) | B27 |
|  | 420 | LPCRIKQII (416-424) | B*5101 |
|  | 433 | KQFINMWQEVGKAMY (421-435) | A2 |
|  | 517, 523 | RAVGMGALIFEFL (511-523) | human |
|  | 615, 616 | GKLICTTTVPWNISWSNK (600-617) | human |
|  | 662 | QELLALDKW (658-666) | Human |
|  | 720 | YSPLSLQTL (712-720) | Cw*0102 |
|  | 744, 747 | **IEEEGGEQDRNRSIRLVNGFLALAWD (741-758)** | Human, A2 |
|  | 760 | LRSLFLFS (760-767) | A*2301 |
|  | 790 | GRRGWEALK (786-794) | B*2705 |
|  | 806 | **LLQYWSQEL (799-807)** | A*0201 |
| CH505 | 4 | RVKEKYQHL (2-10) | B*0801 |
|  | 122 | KLTPLCVTL (121-129) | A*0201 |
|  | 146, 149, 152 | TYNETYNEI (146-154) | Human |
|  | 155 | KNCSFNMTT (155-163) | human |
|  | 175 | KVQKEYAFF* (168-176) | A2 |
|  | 204 | CNTSALTQACPKVTF (196-210) | A*1101 |
|  | 277, 281 | KIAIRSENISNNA (269-281) | Human |
|  | 299, 302, 304 | RPNNNTRKSI (298-307) | B7 |
|  | 336 | SRAKWNNTL (334-342) | B27 |
|  | 349 | TLSQIVTKL (342-349) | A*0201 |
|  | 358 | QFRNKTIVF (352-361) | Cw*0401 |
|  | 371 | PEIVTHS (369-375) | A2 |
|  | 397 | STWNVNGTW (393-400) | B27 |
|  | 416, 419, 420 | LPCRIKQII (416-424) | B*5101 |
|  | 466, 474 | EVFRPGGGDM (466-475) | A*2601 |
|  | 503 | EIKPLGVAPTTTKRR (490-504) | Human |
|  | 643 | NYTSLIYNL (637-645) | Cw*0702 |
|  | 680 | WLWYIKIFI (678-686) | A*0201 |
|  | 719 | YSPLSLQTL (712-720) | Cw*0102 |
|  | 793 | GRRGWEALK (793-759) | B*2705 |
|  | 802 | KYCWNLLQY (794-802) | A*3002 |
| SUMAd34 | 750, 751, 752 | RSSRLVDGFL (744-753) | Human |
| WEAUd212 | 31, 32 | AENLWVTVY (31-39) | B*1801 |
|  | 169 | IRDKVQKEY (165-173) | B27 |
|  | 218 | YCAPAGFAIL (217-226) | Cw*0102 |
|  | 237 | GPCKNVSTVQ (237-246) | B56 |
|  | 298 | VEINCTR (292-298) | A2 |
|  | 336 | SRAKWNNTL (334-342) | B27 |
|  | 354 | QFRNKTIVF (352-361) | Cw*0401 |
|  | 467, 468 | EVFRPGGGDM (466-475) | A*2601 |
|  | 703 | IGLRIVFAVLSVINR (693-707) | A*3303 |
|  | 812 | QELKNSAVSL (805-814) | A*0201 |

**Table S2.** Documented CD8+ T cell epitopes for statistically designated selection sites from 15 15 subjects whose sequence data were obtained from references ([2](#_ENREF_2)), ([3](#_ENREF_3)), ([4](#_ENREF_4)), and ([5](#_ENREF_5)). CD8+ T cell epitope information was obtained from the Los Alamos National Laboratory HIV Molecular Immunology Database (<http://www.hiv.lanl.gov/content/immunology/maps/maps.html>). Epitope sequences in bold represent experimentally confirmed peptides reactive to autologous CD8+ T cell responses in reference ([5](#_ENREF_5)). HLA types in bold represent those match each subject’s HLA types.

References

1. Lee HY, Giorgi EE, Keele BF, Gaschen B, Athreya GS, Salazar-Gonzalez JF, Pham KT, Goepfert PA, Kilby JM, Saag MS, Delwart EL, Busch MP, Hahn BH, Shaw GM, Korber BT, Bhattacharya T, Perelson AS. 2009. Modeling sequence evolution in acute HIV-1 infection. J Theor Biol 261:341-360.

2. Keele BF, Giorgi EE, Salazar-Gonzalez JF, Decker JM, Pham KT, Salazar MG, Sun C, Grayson T, Wang S, Li H, Wei X, Jiang C, Kirchherr JL, Gao F, Anderson JA, Ping LH, Swanstrom R, Tomaras GD, Blattner WA, Goepfert PA, Kilby JM, Saag MS, Delwart EL, Busch MP, Cohen MS, Montefiori DC, Haynes BF, Gaschen B, Athreya GS, Lee HY, Wood N, Seoighe C, Perelson AS, Bhattacharya T, Korber BT, Hahn BH, Shaw GM. 2008. Identification and characterization of transmitted and early founder virus envelopes in primary HIV-1 infection. Proc Natl Acad Sci U S A 105:7552-7557.

3. Salazar-Gonzalez JF, Salazar MG, Keele BF, Learn GH, Giorgi EE, Li H, Decker JM, Wang S, Baalwa J, Kraus MH, Parrish NF, Shaw KS, Guffey MB, Bar KJ, Davis KL, Ochsenbauer-Jambor C, Kappes JC, Saag MS, Cohen MS, Mulenga J, Derdeyn CA, Allen S, Hunter E, Markowitz M, Hraber P, Perelson AS, Bhattacharya T, Haynes BF, Korber BT, Hahn BH, Shaw GM. 2009. Genetic identity, biological phenotype, and evolutionary pathways of transmitted/founder viruses in acute and early HIV-1 infection. J Exp Med 206:1273-1289.

4. Liao HX, Lynch R, Zhou T, Gao F, Alam SM, Boyd SD, Fire AZ, Roskin KM, Schramm CA, Zhang Z, Zhu J, Shapiro L, Program NCS, Mullikin JC, Gnanakaran S, Hraber P, Wiehe K, Kelsoe G, Yang G, Xia SM, Montefiori DC, Parks R, Lloyd KE, Scearce RM, Soderberg KA, Cohen M, Kamanga G, Louder MK, Tran LM, Chen Y, Cai F, Chen S, Moquin S, Du X, Joyce MG, Srivatsan S, Zhang B, Zheng A, Shaw GM, Hahn BH, Kepler TB, Korber BT, Kwong PD, Mascola JR, Haynes BF. 2013. Co-evolution of a broadly neutralizing HIV-1 antibody and founder virus. Nature 496:469-476.

5. Liu MK, Hawkins N, Ritchie AJ, Ganusov VV, Whale V, Brackenridge S, Li H, Pavlicek JW, Cai F, Rose-Abrahams M, Treurnicht F, Hraber P, Riou C, Gray C, Ferrari G, Tanner R, Ping LH, Anderson JA, Swanstrom R, B CC, Cohen M, Karim SS, Haynes B, Borrow P, Perelson AS, Shaw GM, Hahn BH, Williamson C, Korber BT, Gao F, Self S, McMichael A, Goonetilleke N. 2013. Vertical T cell immunodominance and epitope entropy determine HIV-1 escape. J Clin Invest 123:380-393.
